# Assessing the Safety of Carbon Dioxide Extracts of *Acorus calamus* Rhizomes and *Calendula officinalis* Flowers and the Antitussive Activity of the Tablet Dosage Form ‘Exkair’ and Granules ‘Zerp-Ak-Broncho’ Developed on Their Basis

**DOI:** 10.3390/ph19050789

**Published:** 2026-05-18

**Authors:** Galiya Ibadullayeva, Maigul Kizatova, Karlygash Raganina, Meruyert Tleubayeva, Aliya Mamatova, Rauan Botabayeva, Aigerim Karaubaeva, Aktolkyn Ibadullayeva, Aruzhan Darbassova, Lashyn Kiyekbayeva, Rizvangul Ayupova

**Affiliations:** 1 Department of Pharmaceutical Technology, Kazakh National Medical University Named After S.D. Asfendiyarova, Almaty 050000, Kazakhstan; ibadullayeva.g@kaznmu.kz (G.I.); kizatova.m@kaznmu.kz (M.K.); raganina.k@kaznmu.kz (K.R.); tleubayeva.m@kaznmu.kz (M.T.); mamatova.m@kaznmu.kz (A.M.); karaubaeva.a@kaznmu.kz (A.K.); ibadullayeva.a@kaznmu.kz (A.I.); aruzhan_darbasova@mail.ru (A.D.); 2 Department of Pharmaceutical Technology, South Kazakhstan Medical Academy, Shymkent 160001, Kazakhstan; rauana.ex@mail.ru

**Keywords:** acute toxicity, chronic toxicity, antitussive activity, safety assessment, CO_2_ extraction, *Acorus calamus*, *Calendula officinalis*

## Abstract

**Background:** The growing demand for safe and effective phytopharmaceuticals underscores the importance of studying regionally available medicinal plants. *Acorus calamus* L. and *Calendula officinalis* L., widely distributed in the Republic of Kazakhstan, are promising sources of biologically active compounds with significant pharmacological potential. However, the combined use of their CO_2_ extracts remains insufficiently characterised, particularly regarding possible synergistic interactions. Therefore, the development of new dosage forms and their comprehensive pharmacological and toxicological evaluation is a priority in modern pharmaceutical research. **Methods:** Concentrated extracts from *Acorus calamus* rhizomes and *Calendula officinalis* flowers were obtained using precritical CO_2_ extraction. Safety was assessed through acute and chronic toxicity studies in laboratory animals according to standard non-clinical guidelines. Animals received graded doses of the extracts and developed formulations (‘Exkair’ tablets and ‘Zerp-Ak-Broncho’ granules). Clinical condition, mortality, body weight, and behaviour were monitored. Biochemical, haematological, and histopathological analyses were performed. Antitussive activity was evaluated in vivo by measuring oedema inhibition relative to reference drugs. **Results:** The CO_2_ extracts and formulations demonstrated low toxicity and good tolerability, with no mortality or significant adverse effects observed even at high doses. Biochemical and haematological parameters remained within physiological ranges, and histopathological examination revealed no structural alterations in internal organs. Both ‘Exkair’ and ‘Zerp-Ak-Broncho’ exhibited pronounced antitussive activity, confirmed by significant suppression of oedema. This effect is likely associated with the synergistic action of flavonoids, terpenoids, and phenolic compounds. **Conclusions:** The findings indicate that CO_2_ extracts of *Acorus calamus* L. and *Calendula officinalis* L., as well as the developed formulations, possess a favourable safety profile and significant antitussive activity. These results support their further development as phytotherapeutic agents in Kazakhstan.

## 1. Introduction

Over the past several decades, the pharmaceutical industry has primarily focused on synthetic compounds as a major source for the development of new medicinal products. Such compounds are relatively straightforward to synthesise and reproduce, and they are well-suited to high-throughput screening technologies. At the same time, however, there has been a noticeable decline in the number of new drugs reaching the market, which has once again drawn scientific attention to the development of medicines derived from natural sources.

This renewed interest is largely attributed to the wide range of biologically active compounds present in plant materials, as well as their comparatively low toxicity and high biocompatibility with the human body [1,2,3]. According to the World Health Organization, approximately 80% of the global population relies, to some extent, on herbal medicines for the prevention and treatment of various diseases [4]. In this context, the search for novel pharmacologically active substances of natural origin, together with the development of effective and safe medicinal products based on them, remains one of the key priorities of contemporary pharmaceutical science [5,6,7].

Of particular interest are plants containing a complex mixture of secondary metabolites, such as flavonoids, phenolic compounds, terpenoids, saponins, and essential oils, which exhibit pronounced pharmacological activity [8,9,10]. These compounds demonstrate a broad spectrum of biological effects, including anti-inflammatory, antioxidant, antimicrobial, immunomodulatory, and cytoprotective actions [11]. Owing to these properties, medicinal plants are widely utilised in both traditional medicine and the development of contemporary herbal preparations.

In recent years, there has been a growing interest in the investigation of the phytochemical composition of plants, the mechanisms of action of their biologically active constituents, and the development of novel dosage forms based on them. Comprehensive research into medicinal plant materials enables substantiation of their pharmacological value, standardisation of raw materials, and expansion of their potential applications in medical and pharmaceutical practice [12].

One of the promising medicinal plant materials is sweet flag (*Acorus calamus* L.), which is widely distributed across Europe and Asia and has long been used in traditional medicine for the management of gastrointestinal disorders, inflammatory conditions, and infectious diseases, as well as for digestive disturbances, loss of appetite, and flatulence [13,14]. The rhizomes of this plant are extensively employed in the treatment of various conditions, including epilepsy, mental disorders, chronic diarrhoea, dysentery, fever, abdominal tumours, and diseases of the kidneys and liver, as well as rheumatism.

The leaves, rhizomes, and essential oil of *Acorus calamus* exhibit a broad spectrum of biological activities, including antispasmodic and carminative effects, which are summarised in simplified form within this review [15,16]. The pharmacological activity of calamus rhizomes is primarily attributed to their high essential oil content, the main constituents of which include β-asarone, α-asarone, eugenol, and camphene [17,18,19]. In addition, phenolic compounds, flavonoids, bitter principles, and tannins have been identified in this species, contributing to its antioxidant and anti-inflammatory properties [20].

Pharmacological investigations indicate that extracts of *Acorus calamus* possess pronounced antibacterial, anti-inflammatory, gastroprotective, and neuroprotective activities, alongside antispasmodic effects and the ability to stimulate the secretory function of the digestive glands. These findings support the suitability of this plant for the management of gastrointestinal tract disorders. Furthermore, it has been demonstrated that the biologically active constituents of calamus may exert sedative and adaptogenic effects, thereby contributing to the normalisation of the body’s functional state [21,22].

Another valuable source of bioactive compounds is marigold (*Calendula officinalis* L.), which is widely used in pharmaceutical and medical practice due to its anti-inflammatory, antiseptic, antioxidant, and wound-healing properties, as well as its ability to accelerate tissue regeneration, reduce the severity of inflammatory responses, and promote the healing of damaged skin and mucous membranes [23,24]. *Calendula officinalis* L. is a commonly used medicinal plant across Europe, Asia, the United States, and India. It belongs to the family Asteraceae, and it is known under numerous vernacular names [25,26,27].

It has been established that *Calendula officinalis* L. contains a diverse range of secondary metabolites with distinct pharmacological activities, which underlie its therapeutic applications. The principal constituents include triterpenoids, flavonoids, coumarins, quinones, essential oils, carotenoids, and amino acids [28,29]. The flowers of calendula are particularly rich in biologically active compounds, such as flavonoids, triterpene saponins, carotenoids, essential oils, and phenolic acids [30,31,32].

A considerable number of experimental and clinical studies have confirmed the pronounced anti-inflammatory activity of calendula extracts, highlighting this plant as a promising candidate for the development of novel medicinal preparations [33].

In recent years, increasing attention has been devoted to the development of efficient methods for the extraction of biologically active substances from medicinal plant materials. These include microwave-assisted extraction, ultrasonic extraction, enzymatic treatment, and the use of supercritical fluids based on various gases. Such technologies enable an increased yield of target compounds, reduced time and energy consumption, minimisation of thermolabile component degradation, and the elimination of toxic organic solvents [34].

One of the most advanced and promising approaches is supercritical carbon dioxide (CO_2_) extraction. This technique offers several significant advantages over conventional extraction methods, including high selectivity towards target compounds, the absence of toxic organic solvents, and the ability to preserve thermolabile bioactive constituents [35,36,37]. It also allows precise control of extract composition through variation in pressure and temperature, enhances the yield of active components, and ensures the stability and standardisation of the final product.

CO_2_ extracts are widely utilised in the pharmaceutical, cosmetic, and food industries for the development of highly effective and safe preparations based on plant raw materials [38,39]. Supercritical CO_2_ extraction is considered an environmentally friendly technology, as carbon dioxide can be easily removed after the process and reused multiple times within a closed-loop system [40,41,42,43]. Moreover, CO_2_ extracts are characterised by a high degree of purity, stability, and biological activity, making them highly promising for application in the pharmaceutical industry [44].

The use of CO_2_ extracts from medicinal plants opens up new opportunities for the development of innovative herbal medicines with improved pharmacological properties. However, in the development of novel dosage forms based on plant extracts, a comprehensive evaluation of their biological activity and safety is of particular importance. Non-clinical studies allow for the determination of the toxicological characteristics of the investigated substances, the identification of potential adverse effects, and the assessment of their pharmacological efficacy.

Such investigations also contribute to dose optimisation, the selection of appropriate dosage forms, and the prediction of the pharmacokinetic behaviour of active constituents within the body. Contemporary approaches to non-clinical evaluation include in vitro assays for cytotoxicity, antioxidant capacity, and anti-inflammatory activity, as well as in vivo models for the assessment of efficacy, metabolism, and potential systemic toxicity of extracts [45].

Despite a considerable number of studies addressing the pharmacological properties of individual medicinal plants, the combined use of CO_2_ extracts from different plant sources—particularly *Acorus calamus* L. and *Calendula officinalis* L. growing in the Republic of Kazakhstan—remains insufficiently investigated. In particular, the potential synergistic interactions between biologically active compounds in combined herbal preparations are of significant scientific interest. In this regard, the development of new dosage forms containing CO_2_ plant extracts, alongside their comprehensive pharmacological and toxicological evaluation, represents an important task in contemporary pharmaceutical science.

The aim of this study was to investigate the safety of CO_2_ extracts obtained from the rhizomes of *Acorus calamus* L. and the flowers of *Calendula officinalis* L., growing in the Republic of Kazakhstan, as well as to evaluate the antitussive activity of tablet and granule dosage forms, provisionally named ‘Exkair’ and ‘Zerp-Ak-Broncho’, developed on the basis of these extracts.

## 2. Results

### 2.1. GC-MS Results for Carbon Dioxide Extracts of Calendula officinalis (Table 1, Figure 1) and Acorus calamus (See Table S1, Figure S1)

The CO_2_ extract was analysed by gas chromatography with mass spectrometric detection (7890A/5975C).

**Table 1:** Results of GC–MS analysis of the CO_2_ extract of *Calendula officinalis* L.

№	Retention Time (min)	Compounds	Probability of Identification (%)	Percentage Content (%)
1	7.652	2-methylbutanoic acid	91	2.95
2	11.417	Hexanoic acid	79	1.38
3	13.324	1-methyl-2-(propan-2-yl)benzene	92	0.97
4	13.616	1,8-cineole	91	0.85
5	17.467	5-methyl-2-(propan-2-yl)cyclohexan-1-one	94	1.57
6	17.753	5-methyl-2-(propan-2-yl)cyclohexan-1-one	91	0.46
7	18.219	4-methyl-1-(propan-2-yl)cyclohex-3-en-1-ol	92	1.15
8	21.213	1-(propan-2-yl)-4-methylidenebicyclo [3.1.0]hexan-3-yl acetate	89	0.93
9	22.985	1,4-dimethyl-7-(propan-2-yl)bicyclo [4.4.0]dec-1-ene	92	0.62
10	23.832	2,6-dimethyl-8-(propan-2-yl)tricyclo [5.3.1.0^1,5^]undec-2-ene	94	1.71
11	26.134	Muurola-4(15),5-diene	92	0.72
12	26.389	γ-Muurolene	89	2.01
13	26.85	1,1,4,7-tetramethyl-1,2,3,4,5,6,7,7a-octahydro-1H-cyclopropa[e]azulene	84	0.71
14	26.964	α-Muurolene	92	4.31
15	27.377	7-methyl-4-methylidene-1-(propan-2-yl)-octahydronaphthalene	91	7.65
16	27.446	1-(propan-2-yl)-4,7-dimethyl-1,2,3,5,6,8a-hexahydronaphthalene	90	15.45
17	27.563	Calamenene	90	1.76
18	27.817	4,4,7a-trimethyl-5,6,7,7a-tetrahydro-2-benzofuranone	87	1.14
19	27.936	1-(propan-2-yl)-4,7-dimethyl-1,2,3,4,4a,5,6,8a-octahydronaphthalene	94	1.30
20	29.464	decahydro-1,1,4,7-tetramethyl-1H-cyclopropa[e]azulen-4-ol	87	0.44
21	29.879	cedr-8-en-3-ol	89	1.81
22	30.161	cub-2-en-11-ol	90	0.69
23	30.509	muurol-4-en-1-ol	93	5.22
24	30.786	cadin-4-en-1-ol	94	6.50
25	32.559	4-hydroxy-7-(propan-2-yl)-4-methyloctahydro-1H-inden-1-yl ethanone	89	1.50
26	33.148	6-hydroxy-4,4,7a-trimethyl-5,6,7,7a-tetrahydro-2-benzofuranone	87	0.72
27	34.278	6,10,14-trimethylpentadecan-2-one	67	2.31
28	34.72	Hexacosane	89	31.94
29	35.955	Hexadecanoic acid	83	0.64
30	37.738	Heneicosane	87	0.60

**Figure 1:**
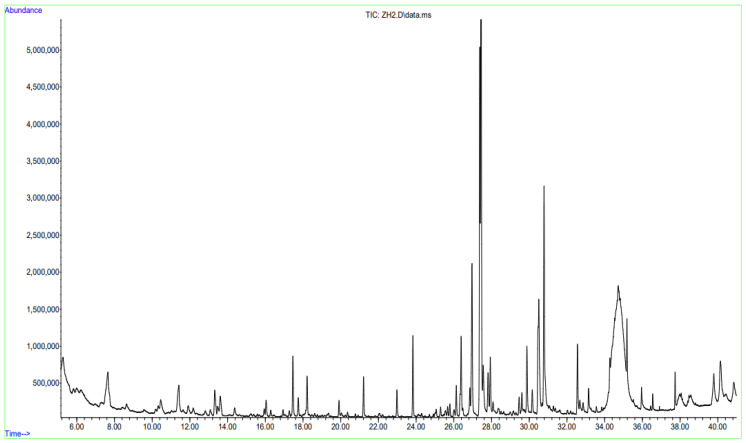
Chromatogram of the CO_2_ extract of *Calendula officinalis* L.

### 2.2. Assessment of the Acute and Chronic Toxicity of CO_2_ Extracts

Following administration of the CO_2_ extracts, no impairment of motor coordination, dyspeptic disorders, weight loss, or animal deaths were recorded. All reflexes were preserved (see Table 2).

In conclusion, it can be stated that the carbon dioxide (CO_2_) extracts exhibited no toxic effects in the experimental animals and are safe when administered as a single dose. The observational results demonstrated that no fatalities occurred among the animals throughout the entire experimental period. Moreover, no pronounced signs of intoxication were observed in the test animals, including convulsions, depression of motor activity, impaired coordination of movements, or marked behavioural changes. The general condition of the animals remained satisfactory: the coat retained its normal sheen and density, and the mucous membranes were of normal colour.

The results of the chronic toxicity study revealed no coordination disorders, dyspeptic disturbances, weight loss, or mortality among the animals. All reflexes were preserved (Table 3). Consequently, it was confirmed that the carbon dioxide (CO_2_) extract did not exert any toxic effects on the experimental animals and is safe upon repeated administration.

Dynamic observations demonstrated that body weight parameters in the experimental groups did not differ from those in the control group and were characterised by a steady increase throughout the entire study period. This finding indicates the absence of any adverse effect of the investigated CO_2_ extracts on the overall metabolic status of the organism (see Table 4).

As indicated above, during the experiment, a positive trend in the body weights of the mice was observed, i.e., weight gain within the normal range, throughout the study.

Thus, the data obtained suggest that the LD_50_ values of the CO_2_ extracts under investigation exceed 2500 mg/kg body weight, indicating a low level of acute toxicity of these substances. According to the generally accepted toxicological classification, such indicators allow the investigated compounds to be classified as substances with low toxicity.

The results obtained are consistent with data from previous studies, which also reported the low toxicity of *Acorus calamus* and *Calendula officinalis* CO_2_ extracts, attributable to their natural origin and the presence of biologically active compounds with high biocompatibility [1,2].

Following the conclusion of the experiment, five white mice were euthanised in order to identify the organs most susceptible to toxicity and to determine the weight of the internal organs. The animals’ internal organs were sent to the pathological laboratory.

Analysis of haematological tests revealed minor deviations in the counts of erythrocytes, leucocytes, and haemoglobin (see Table 5).

At the conclusion of the chronic toxicity assessment of carbon dioxide (CO_2_) extracts, macroscopic and microscopic analyses of the internal organs were performed. Macroscopic examination of the internal organs of mice in all study groups revealed no evidence of toxic effects or pathological alterations.

Macroscopic analysis. Upon dissection of the experimental animals, it was observed that the colour, consistency, and anatomical–topographical parameters of the internal organs within the thoracic and abdominal cavities remained unchanged. The anatomical structure and positioning of the organs were normal; traces of clear fluid were detected in the thoracic cavity, and the blood vessels were well perfused.

Following the determination of the weights of the internal organs, no statistically significant changes were observed. The results of these observations are presented in Table 6.

Pathomorphological picture. The coat of the experimental white mice was clean and glossy; no areas of alopecia were observed. The heart volume and shape remained unchanged. The cardiac muscle exhibited a doughy consistency and was brown in colour. The surface of the lungs was light red. The bronchial mucosa was smooth and light red in colour; no signs of haemorrhage were observed (see Supplementary File S1, Figure S2).

The gastric mucosa was light red in colour, with no signs of haemorrhage or ulceration detected. The mucous membranes of the small and large intestines were also light red, with no evidence of bleeding or ulcerative lesions. The liver retained its normal shape and size; the hepatic capsule was thin and transparent. The liver had a doughy consistency and was dark red in colour.

The kidneys showed no visible alterations and were light brown in colour. Their surface was smooth, and on sectioning, the cortical and medullary regions were clearly distinguishable. The spleen was reddish-brown in colour, with a smooth surface and doughy consistency.

No pathological changes were detected during macroscopic examination of the internal organs in groups 2, 3, 4, and 5 in the study of the chronic toxicity of carbon dioxide (CO_2_) extracts.

### 2.3. Assessment of Local Irritant Effects

An important stage in the non-clinical safety assessment of new medicinal products is the investigation of local irritant potential. During the experiment, the test preparations were applied to the skin of laboratory animals, followed by observation for the development of any inflammatory reactions.

No conjunctival hyperaemia, inflammation, or lacrimation was observed. The general condition of the animals remained satisfactory throughout the study.

### 2.4. Investigation of Allergenic Properties

No allergic reactions were detected during the study of CO_2_ extracts of *Acorus calamus* rhizomes and *Calendula officinalis* flowers.

Following conjunctival administration of the solution, no ocular hyperaemia, inflammation, or lacrimation was observed. The condition of the animals remained satisfactory throughout. At 15 min and at 24–48 h post-administration, no signs of hypersensitivity were detected, which were assessed according to the following scale:

1—slight redness of the lacrimal duct;
2—redness of the lacrimal duct extending towards the eyelid;
3—redness of the entire eyelid.

The CO_2_ extracts investigated did not induce any allergic reactions.

The results of the study demonstrated no pronounced signs of local irritation. No hyperaemia, oedema, or other signs of an inflammatory response were observed at the site of application. The skin remained in a physiological state, indicating good tolerability of the substances under investigation.

Overall, the findings allow us to conclude that CO_2_ extracts of *Acorus calamus* and *Calendula officinalis* do not exert an irritating effect on the skin and may be considered safe for both topical and oral use.

### 2.5. Antitussive Activity of the Tested Preparations

The efficacy of the test preparations was evaluated based on the reduction in the frequency of coughing episodes. The results were expressed as a percentage relative to the untreated control group, which was taken as 100%.

The antitussive agent Libexin was used as the reference drug and was administered as a suspension at a dose of 100 mg/kg body weight.

The results of the study allow the efficacy of the authors’ antitussive preparations to be evaluated in comparison with standard therapy, and enable the identification of promising directions for the development of new medicinal products aimed at suppressing the cough reflex in inflammatory diseases of the respiratory tract.

Experimental Groups III, IV, V, and VI received the standard drug Libexin at various dosages. Groups VII, VIII, VIIa, and VIIIa were treated with the authors’ preparations in the form of tablets and granules as therapeutic agents.

## 3. Discussion

### 3.1. GC-MS Analysis of Calendula officinalis L.

The analysis of the chemical composition of *Calendula officinalis* using GC-MS (Table 1) revealed a complex profile predominantly composed of terpenes, oxygen-containing derivatives, and aliphatic hydrocarbons. The confidence level of identification for most compounds ranged from 84% to 94%, indicating a high degree of reliability in the obtained results. Similar levels of analytical accuracy have been reported in recent studies of plant extracts and essential oils [46,47,48,49].

Hexacosane (31.94%) was identified as the dominant component of the extract. The predominance of high-molecular-weight aliphatic hydrocarbons has previously been associated with lipophilic and waxy fractions of plant material, particularly in samples obtained by supercritical CO_2_ extraction [27,50]. Such compounds play an important role in the protective and stabilising functions of plant matrices.

The second most abundant compound, 1-isopropyl-4,7-dimethyl-1,2,3,5,6,8a-hexahydronaphthalene (15.45%), belongs to sesquiterpene hydrocarbons, confirming the pronounced terpenoid nature of the investigated raw material. According to recent reviews, Calendula officinalis contains a wide range of terpenes, including mono-, sesqui-, and triterpenes, which are responsible for its pharmacological activity [51,52].

Other significant constituents included naphthalene derivatives (7.65%), α-cadinol (6.50%), and τ-muurolol (5.22%). These oxygenated sesquiterpenes are well known for their antimicrobial, anti-inflammatory, and antioxidant activities, as confirmed by recent experimental studies [53,54,55]. The presence of α-muurolene further supports the sesquiterpene-rich composition of the sample.

Fatty acids and their derivatives, including 2-methylbutanoic acid, hexanoic acid, and hexadecanoic acid, were also detected and may contribute to both the biological activity and organoleptic properties of the extract. Recent studies indicate that such compounds are involved in anti-inflammatory and antimicrobial mechanisms [52].

Minor constituents included monoterpenes such as o-cymene and eucalyptol, which are characteristic of essential oils and are associated with bronchodilatory and anti-inflammatory effects [51].

The presence of cyclic ketones, alcohols, and benzofuran compounds suggests active secondary metabolism and possible biosynthetic transformations of terpenes. Recent metabolomic studies confirm that *Calendula officinalis* contains a diverse range of secondary metabolites, including phenolics, carotenoids, and terpenoids [52].

Overall, the chemical profile is characterised by a predominance of sesquiterpenes and high-molecular-weight hydrocarbons in combination with oxygen-containing bioactive compounds. This composition reflects the complex phytochemical nature of *Calendula officinalis* and supports its significant pharmacological potential, particularly in terms of anti-inflammatory and antioxidant activity [49,51,52].

The high proportion of hexacosane warrants further consideration. Its elevated content may be attributed to both intrinsic properties of the plant material and technological factors associated with supercritical CO_2_ extraction, which preferentially isolates lipophilic compounds [50].

In conclusion, the present study confirms the multi-component composition of *Calendula officinalis* and is consistent with recent literature data on the chemical composition and biological activity of this plant [49,51,52]. These findings provide a solid foundation for further pharmacological and technological investigations.

### 3.2. Acute Toxicity

During the experiments, the acute toxicity of CO_2_ extracts derived from the rhizomes of sweet flag (*Acorus calamus* L.) and the flowers of marigold (*Calendula officinalis* L.) was evaluated. The investigated CO_2_ extracts were administered orally to laboratory animals at doses of 300, 500, 900, and 2500 mg/kg body weight.

Following administration, no impairments in motor coordination, dyspeptic disorders, body weight loss, or mortality were observed. All reflexes remained intact (see Table 3).

### 3.3. Chronic Toxicity

**Figure 2:**
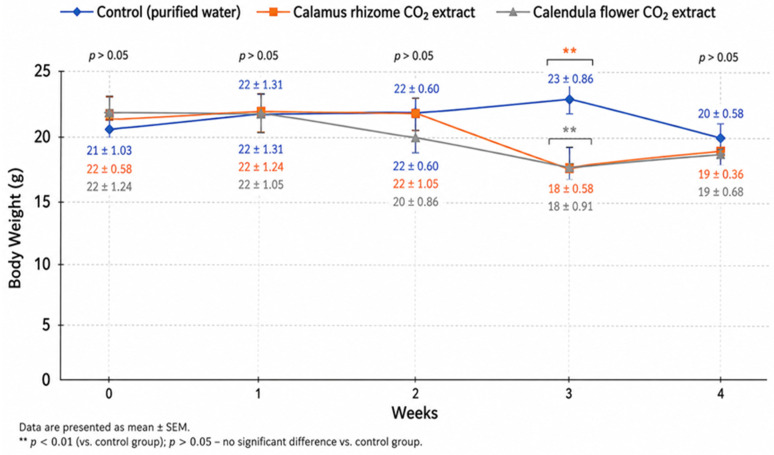
Changes in body weight in white mice after 4 weeks of administration of the test substance.

According to Figure 2, the control group (purified water) exhibited stable body weight dynamics throughout the experiment. Body weight increased gradually from 21 g to 23 g by the third week, followed by a slight decrease to 20 g in the fourth week. Overall, these fluctuations remained within physiological limits, indicating the absence of stress or toxic effects, and are consistent with standard laboratory animal growth patterns reported in recent studies [53].

In the group receiving the CO_2_ extract of *Acorus calamus* rhizomes, body weight remained stable during the first two weeks (22 g); however, a marked and statistically significant decrease to 18 g was observed in the third week (*p* < 0.01). In the fourth week, a slight recovery to 19 g was noted, although baseline values were not fully restored. Such transient weight loss may reflect metabolic adaptation or reduced food intake, as previously described for phytochemical-rich extracts that influence energy metabolism and appetite regulation [54,55].

A similar pattern was observed in the group treated with the CO_2_ extract of *Calendula officinalis* flowers. Following relatively stable values during the initial weeks (22–20 g), a significant decrease to 18 g occurred in the third week (*p* < 0.01), followed by partial recovery to 19 g by the fourth week. These findings are consistent with recent reports indicating that plant-derived bioactive compounds, including terpenoids and flavonoids, may induce transient metabolic changes without causing persistent toxicity [56].

Comparative analysis indicates that both plant-derived CO_2_ extracts elicited a similar biological response, characterised by a reduction in body weight during the third week, followed by a tendency towards recovery. The statistically significant differences observed specifically in the third week suggest a transient rather than cumulative effect of the treatment.

Overall, the data obtained suggest that the investigated CO_2_ extracts do not exert pronounced toxic effects, as evidenced by the partial restoration of body weight. However, they may temporarily influence the physiological state of the animals, particularly parameters related to metabolism and body weight regulation. Similar reversible effects have been reported in recent toxicological and pharmacological studies of plant extracts, where moderate weight fluctuations were associated with adaptive physiological responses rather than systemic toxicity [57].

### 3.4. Histological Studies

Histological examination of the lungs of white mice administered the tested carbon dioxide (CO_2_) extract of *Acorus calamus* rhizomes revealed clear alveolar spaces and an intensely stained bronchial epithelium. In isolated areas, minor fresh haemorrhages were observed, most likely associated with the euthanasia procedure (decapitation). No signs of circulatory disturbances, oedema, or inflammatory infiltration were detected.

Overall, no pathological alterations were identified in the lung tissue of animals treated with the CO_2_ extract, indicating the absence of pulmonary toxicity under the studied conditions. A similar absence of structural pulmonary damage following administration of plant-derived extracts has been reported in recent toxicological evaluations of herbal preparations [58].

Histological examination of the kidneys of white mice treated with the CO_2_ extract of *Acorus calamus* rhizomes revealed uniform staining of the tubular epithelium, with nuclear structures exhibiting normal chromatin uptake. The histoarchitectural organisation of the renal tissue was fully preserved. No haemorrhage, oedema, or degenerative changes were detected. These findings indicate preserved renal integrity and the absence of nephrotoxic effects, consistent with recent reports on the renal safety profile of phytochemical-rich CO_2_ extracts [58].

Similarly, examination of the liver (see Supplementary File S1) demonstrated well-preserved hepatocyte morphology, normal staining intensity, and intact hepatic lobular architecture. Hepatocytes were arranged in a typical radial pattern, and no signs of necrosis, haemorrhage, or inflammatory infiltration were observed. These results suggest that the investigated CO_2_ extract does not induce hepatocellular damage under the experimental conditions.

Overall, macroscopic and histological examinations demonstrated that oral administration of the CO_2_ extract of *Acorus calamus* rhizomes does not induce general pathological or organ-specific destructive changes in the lungs, kidneys, or liver of experimental animals. The absence of toxic effects suggests a favourable preliminary safety profile under the applied dosing regimen. These findings are consistent with recent subacute toxicity studies of plant-derived extracts, which likewise report a lack of significant histopathological alterations in major organs [58].

### 3.5. Antitussive Activity of the Studied Preparations

During the investigation of the expectorant activity of carbon dioxide (CO_2_) extracts obtained from the rhizomes of marsh calamus and the flowers of medicinal marigold, it was established that the developed dosage forms—‘EXKAIR’ tablets and ‘ZERP-AK BRONCHO’ granules—exhibited pharmacological activity at doses of 3, 5, 9, and 25 mg/kg body weight in guinea pigs.

As shown in Table 7, administration of ‘EXKAIR’ at a dose of 3 mg/kg resulted in an expectorant activity of 1.22%. In contrast, the reference drug Libexin, administered at the same dose, demonstrated an activity of 41.85%, which was approximately 34.3 times higher than that of the test preparation.

At a dose of 5 mg/kg, the expectorant activity of ‘EXKAIR’ increased to 14.16%, whereas Libexin showed an activity of 35.83%, which was approximately 2.53 times higher than that of the test preparation.

At a dose of 9 mg/kg, the activity of ‘EXKAIR’ reached 31.42%, while Libexin exhibited an activity of 30.55%. Thus, at this dose, the efficacy of the test preparation was slightly higher than that of the reference drug.

The most pronounced pharmacological effect of ‘EXKAIR’ was observed at a dose of 25 mg/kg, at which its expectorant activity reached 57.27%. In comparison, the reference drug Libexin, administered at the same dose, demonstrated an activity of 24.19%, indicating that the efficacy of the test preparation was more than 2.36-fold higher.

Statistical analysis of the results showed that at a dose of 9 mg/kg, ‘EXKAIR’ exhibited activity exceeding that of Libexin by 1.02-fold and that of Mucaltin by 1.04-fold (*p* < 0.001). The obtained data indicate a dose-dependent pharmacological effect: as the dose of ‘EXKAIR’ increases, its expectorant activity correspondingly rises. Furthermore, the LD_50_ value for ‘EXKAIR’ was determined to be 0.45.

Table 8 presents the correlation coefficients for ‘EXKAIR’, Libexin, and Mucaltin. According to the data presented in Table 9, the granulated dosage form ‘ZERP-AK BRONCHO’, at a dose of 25 mg/kg, demonstrated an expectorant activity of 58.37%. This value exceeds the efficacy of Libexin by 2.41-fold and that of Mucaltin by 1.58-fold.

The results obtained indicate the high pharmacological activity of the investigated dosage forms and confirm the potential of CO_2_ extracts from the rhizomes of *Acorus calamus* and the flowers of *Calendula officinalis* for the development of novel preparations with expectorant properties.

After completion of the experiment and euthanasia of the animals, the bronchi were fixed in neutral formalin and subsequently processed through a graded series of alcohol solutions of increasing concentration. Sections were prepared from paraffin-embedded blocks and stained with haematoxylin and eosin for morphological analysis. The prepared specimens were examined microscopically to assess structural changes in the bronchi.

Animals in the third group received the reference drug Libexin at a dose of 3 mg. Morphological examination revealed pronounced alterations in the structure of the large bronchi, associated with oedema of the bronchial walls. Accumulation of fluid between the cartilaginous elements disrupted their orderly arrangement. As a result, the bronchial lumen was narrowed and acquired a characteristic “hourglass” configuration (see Figure S3).

The epithelium was displaced towards the bronchial lumen, leading to partial denudation of the mucosa. Numerous inflammatory infiltrates were observed within the bronchial wall (see Figure S3). Infiltration of the bronchial epithelium was clearly evident, and inflammatory changes were also noted in the cartilage (see Figure S4).

In animals of the third group, thickening of the interalveolar (interstitial) structures of the lung tissue was observed. These changes were primarily associated with venous congestion and increased cellular proliferative activity. The alveolar spaces were reduced, and marked peribronchial infiltration was observed surrounding the bronchi (see Figure S5).

Morphological examination revealed well-defined peribronchial infiltration in the medium and large bronchi, accompanied by venous congestion. The bronchial epithelium exhibited papillary proliferation into the lumen, resulting in its narrowing. In some sections, fragments of cartilaginous tissue were also detected (see Figure S6). These changes are consistent with the development of bronchitis and peribronchial inflammation, ultimately progressing to interstitial pneumonia.

In the fourth group, treated with Libexin at a dose of 5 mg, histological examination revealed focal desquamation of the epithelium in the large bronchi. The bronchial walls were thickened due to oedema and venous congestion, while peribronchial inflammatory infiltration persisted (see Figure S7). Alterations were also observed in the cartilaginous tissue, where vacuoles containing cytoplasmic fluid were present within chondrocytes, indicating oedema and a cellular response to inflammation.

In the lung tissue at the level of the medium bronchi, pronounced papillary proliferation of the epithelium resulted in significant narrowing of the bronchial lumen. The vessels surrounding the bronchi were engorged with blood, and the bronchial walls remained thickened. Peribronchial inflammatory infiltration extended along the entire length of the bronchi (see Figure S7).

The inflammatory process in the lungs assumed a focal character. In isolated areas, thinning of the interalveolar septa was observed, with no oedema or blood-filled vessels detected. The number of cellular elements was reduced. Papillary proliferation of the epithelium of the medium and small bronchi persisted, whilst peribronchial inflammatory infiltrates were localised around the bronchi, forming dense inflammatory foci (see Figure S8).

Peribronchial inflammatory infiltrates persisted in the large bronchi, with the epithelium exhibiting papillary proliferation (see Figure S9). Due to the narrowing of the mucosal layer, the bronchial lumen was distorted. The inflammatory process in the lungs remained focal in nature; however, the proliferative activity of the epithelium persisted, manifested as peribronchial infiltration. Marked oedema of epithelial cells was also observed (see Figure S10).

Figure S6 shows papillary proliferation of the epithelium and focal peribronchial infiltration at ×100 magnification, stained with haematoxylin and eosin. Figure S7 shows a large bronchus with focal epithelial proliferation and peribronchial infiltration at ×100 magnification, unstained.

Histological analysis revealed that focal epithelial proliferation in the large bronchus persisted; signs of oedema had somewhat decreased, whilst peribronchial infiltration remained at a similar level (see Figure S11). These findings indicate that treatment of inflammatory processes in the lungs and bronchi with standard preparations at various dosages contributes to a reduction in the severity of inflammatory changes.

Morphological examination of animals in the seventh group, which received the authors’ preparations in the form of 9 mg tablets, demonstrated preservation of the structure of the large bronchi. The epithelium appeared smooth and uniform along the entire length of the bronchus; however, focal peribronchial infiltration persisted (see Figure S12).

The epithelium was uniformly smooth throughout; however, focal peribronchial inflammatory infiltration persisted (see Figure S14). In the lung tissue, the interalveolar septa were thin; thickening was observed only in the small and medium-sized bronchi. The bronchial epithelium was single-layered and shortened in some areas, with focal swelling of the walls and congested blood vessels (see Figure S12).

Histological examination of animals in the eighth group following administration of the drug in the form of a 25 mg tablet showed that the lumen of the large bronchus remained patent. A small focus of inflammatory infiltration was observed in the bronchus (see Figure S13). Due to oedema, the bronchial walls were thickened, while the epithelium remained fully preserved. Multilayered epithelium formed as a result of basal cell proliferation was also preserved. The histological structure of the cartilaginous plate remained unchanged: the cytoplasm was clearly defined, and the cells had round nuclei. No inflammatory infiltration was detected in this region. The muscular layer was preserved (see Figure S15). However, signs of peribronchial pneumonia persisted in the lung tissue.

Focal sclerosis of the bronchial walls was detected in the medium bronchi, and signs of peribronchial infiltration were observed in certain areas.

Microscopic examination of animals in Group 8A, which received 25 mg granules, revealed the following histopathological changes: focal areas of pneumonia were observed within the lung parenchyma. In the middle-sized bronchi, peribronchial sclerosis and sparse peribronchial inflammatory infiltrates were detected. In some regions, the bronchial epithelium was single-layered and preserved, whereas in others, papillary epithelial proliferations extending into the lumen were noted (see Figure S16).

In the large bronchi, signs of epithelial hypertrophy and subepithelial inflammatory infiltration were observed, while the epithelial lining remained largely intact (see Figure S17). Overall, inflammatory changes in the lungs and bronchi were attenuated and were accompanied by the formation of foci of reparative regeneration, restoration of the bronchial epithelium, and partial resolution of peribronchial sclerosis (see Figure S18).

The reduction in inflammatory processes was supported by the presence of regenerative foci. Restoration of the bronchial epithelial layer and regression of peribronchial sclerosis indicate a favourable response to the administered treatment.

As is well established, the cardinal features of inflammation include oedema, exudation, cellular proliferation, and tissue alteration. In the present study, morphological manifestations of the inflammatory response were identified in the experimental animals, including subepithelial and intercartilaginous inflammatory infiltration, as well as accumulation of inflammatory exudate. Exudation was associated with an increased presence of inflammatory cells. As a consequence of the inflammatory process, narrowing of the bronchial lumen was observed, along with epithelial alteration and, in some areas, partial epithelial desquamation.

Guinea pigs were used in the experiment and divided into eight groups:

Group I—control group.

Groups II–V received the standard preparation Libexin at various dosages.

Groups VII, VIII, VIIa, and VIIIa were treated with the author’s preparation ‘Exkair’ in tablet form.

The histopathological semi-quantitative assessment is presented in Table 10 below (see Supplementary File S2).

**Figure 3:**
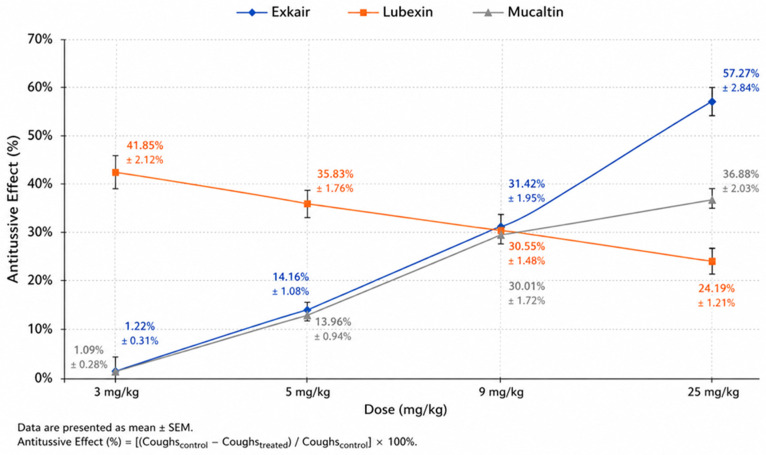
Comparative analysis of the antitussive activity of the agents.

Over the course of 7 days, the experimental groups were administered the corresponding preparations, Libexin and Exkair, orally in order to assess their effect on inflammatory processes in the respiratory tract (see Table 11, Figure 3).

Experimental studies were conducted on animals treated with a 10% formalin solution and high-concentration alcohol solutions. Tissue sections were prepared from paraffin-embedded blocks, stained with haematoxylin and eosin, and subsequently examined under light microscopy.

Group III animals received Libexin at a dose of 3 mg. Morphological examination revealed inflammatory changes in the walls of the large bronchi. Accumulation of fluid beneath the cartilage disrupted its normal architecture, while the bronchial lumen was narrowed and deformed, acquiring an ‘hourglass’ configuration. Epithelial desquamation, subepithelial inflammatory infiltration, and degenerative changes in the cartilaginous tissue were observed. The alveolar septa were also thickened.

These findings are consistent with the development of bronchitis accompanied by interstitial pneumonia.

Group IV—Animals received Libexin at a dose of 5 mg. Foci of epithelial desquamation were observed in the large bronchi. Oedema and vascular congestion resulted in thickening of the bronchial walls. Peribronchial inflammatory infiltration persisted around the bronchi. The cartilaginous tissue also showed inflammatory changes, with vacuoles containing cytoplasmic fluid detected within the cytoplasm of chondrocytes.

Group V—Animals received Libexin at a dose of 9 mg. The inflammatory processes became focal in nature. In isolated areas of the lungs, thinning of the alveolar septa was observed. Venous engorgement and oedema were absent, and the number of inflammatory cells was reduced. Peribronchial inflammatory infiltration persisted around the bronchi. The epithelium exhibited papillary proliferation, resulting in narrowing and deformation of the bronchial lumen.

Group VI—Animals received Libexin at a dose of 20 mg. Pulmonary inflammation persisted; however, epithelial proliferation was observed in the form of peribronchial infiltration. Subepithelial inflammatory changes remained, and infiltration of cells within the large bronchi was noted. Histological analysis demonstrated a reduction in inflammatory signs, confirming the positive effect of the reference preparation.

Group VII—Animals received ‘Exkair’ tablets at a dose of 9 mg. The large bronchi were intact, and the epithelium appeared smooth and uniform. Focal inflammatory infiltrates persisted. The alveolar septa of the lungs were thin, with thickening observed only in the medium-sized bronchi. The epithelium was predominantly single-layered, and the blood vessels were engorged.

Group VIII—Animals received ‘Exkair’ tablets at a dose of 25 mg. The structure of the large bronchi was preserved, and the bronchial lumen remained unobstructed. The epithelium was intact, with only minor foci of peribronchial inflammatory infiltration observed.

Group VIIa—Animals received ‘ZERP-AK-BRONCHO’ granules at a dose of 9 mg. The walls of the large bronchi were thickened due to inflammatory processes. The epithelium was preserved, with active basal cells, and the multilayered epithelial structure remained intact. The cartilaginous framework was unchanged, with clearly defined cytoplasm and round nuclei, and no inflammatory infiltration. The muscular layer was preserved. Foci of peribronchial pneumonitis were observed in the lungs, while sclerosis and peribronchial infiltration were detected in the medium-sized bronchi.

Group VIIIa—Animals received ‘ZERP-AK-BRONCHO’ granules at a dose of 25 mg. Foci of pneumonitis were detected in the lungs. Peribronchial sclerosis and inflammatory infiltrates were observed in the medium-sized bronchi. Subepithelial inflammatory infiltration was present in the large bronchi, while the epithelium remained intact.

A comparative analysis of antitussive efficacy demonstrates dependent patterns for all three preparations:

1. Exkair:

Exhibits a marked dose-dependent increase in antitussive activity.

The effect increases from 1.22% (3 mg/kg) to 57.27% (25 mg/kg).

This indicates a progressive pharmacological response and points to high therapeutic potential with increasing dose.

2. Libexin:

It is most effective at the minimum dose (41.85% at 3 mg/kg).

However, as the dose increases, efficacy decreases, reaching 24.19% at 25 mg/kg.

This inverse relationship may be due to receptor saturation, desensitisation, or an optimal effect at low doses.

3. Mucaltin: Demonstrates a moderate dose-dependent increase in activity.

The effect increases from 1.09% to 36.88%. Although the drug is inferior to Exkair at high doses, it is characterised by a stable and predictable pharmacological action.

Overall comparison: At a low dose (3 mg/kg), Libexin demonstrated the greatest efficacy. At medium doses (5–9 mg/kg), Exkair and Mucaltin approached the efficacy of Libexin, with Exkair showing slightly higher activity at 9 mg/kg. At a high dose (25 mg/kg), Exkair exhibited the greatest efficacy (see Figure 3).

Mucaltin occupied an intermediate position, whereas Libexin showed the lowest efficacy at this dose level.

Thus, Exkair presents the most promising pharmacological profile due to its pronounced dose-dependent efficacy and maximal therapeutic effect. Libexin appears more suitable for use at low doses; however, the reduction in efficacy with increasing dose limits its therapeutic flexibility. Mucaltin demonstrates a moderate and stable effect but does not reach the efficacy level of Exkair.

Morphological assessment confirmed that maximal efficacy was achieved with Exkair tablets at a dose of 9 mg (31.42% efficacy) and with ‘ZERP-AK-BRONCHO’ granules at a dose of 25 mg (58.37% efficacy). The use of lower doses of Exkair (3 and 5 mg) and ‘ZERP-AK-BRONCHO’ granules was less effective.

Experimental data demonstrated that both preparations contribute to a reduction in exudation, restoration of cartilaginous tissue, and regeneration of the epithelium. The active constituents—carbon dioxide (CO_2_) extracts of calamus rhizomes and marigold flowers, as well as ascorbic acid—are responsible for the observed therapeutic effect.

**Figure 4:**
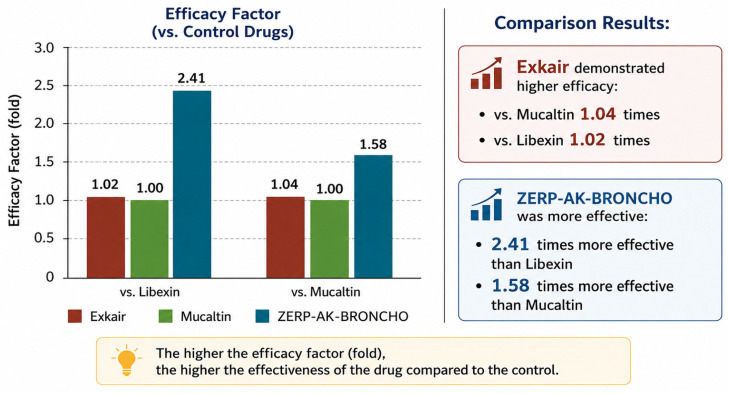
Comparative evaluation of efficacy relative to the reference drug.

Exkair demonstrated higher efficacy than Mucaltin by a factor of 1.04 and than Libexin by a factor of 1.02. ‘ZERP-AK-BRONCHO’ was 2.41 times more effective than Libexin and 1.58 times more effective than Mucaltin (see Figure 4).

The comparative analysis demonstrates that both investigated formulations exhibit higher antitussive efficacy compared with the reference drugs.

Exkair shows only a slight improvement over the standard therapies, exceeding Mucaltin by a factor of 1.04 and Libexin by 1.02. These values indicate a marginal increase in efficacy, suggesting that Exkair has a comparable therapeutic activity to conventional antitussive agents.

In contrast, ‘ZERP-AK-BRONCHO’ demonstrates a markedly superior effect. Its efficacy is 2.41 times higher than that of Libexin and 1.58 times higher than that of Mucaltin. This substantial difference indicates a pronounced pharmacological advantage and suggests a stronger antitussive potential.

Overall, the results indicate that while Exkair provides a modest improvement over existing treatments, ‘ZERP-AK-BRONCHO’ exhibits significantly enhanced efficacy and may be considered a more promising candidate for further pharmacological development.

The semi-quantitative evaluation of histopathological changes demonstrated clear and statistically relevant differences between the experimental groups, reflecting the biological response of bronchopulmonary tissue to the investigated substances. Under control conditions, the lung tissue architecture remained largely intact, with minimal or no pathological alterations and correspondingly low histopathological scores across all evaluated parameters (Table 10).

These findings are consistent with the normal morphological structure of rodent lung tissue described in standard toxicological references, where an intact epithelium and the absence of inflammatory infiltration are considered baseline features [59].

In the experimental groups exposed to the investigated compounds, a dose-dependent increase in the severity of morphological alterations was observed. Such dose–response relationships are widely recognised as a key criterion in toxicological interpretation within preclinical safety assessment. The most frequently observed changes included epithelial oedema and infiltration by inflammatory cells, ranging from mild to moderate severity. These alterations are characteristic of early inflammatory responses mediated by cytokine activation and increased vascular permeability, as described in models of chemically induced airway irritation [60].

In several cases, epithelial hyperplasia was detected, suggesting adaptive and compensatory mechanisms of the airway mucosa in response to repeated or prolonged exposure to bioactive substances. Similar regenerative and proliferative responses have been reported in experimental studies involving inhaled phytochemical and irritant compounds. More pronounced structural damage, including bronchial lumen deformation, vascular congestion, and peribronchial inflammation, was observed in selected groups and corresponded to moderate-to-severe tissue injury. These findings are consistent with established criteria for histopathological grading of pulmonary toxicity, where vascular and peribronchial changes are regarded as indicators of progressive inflammatory damage [59,60].

Overall, the results confirm that the investigated compounds induce histopathological alterations of varying severity in bronchopulmonary tissue, reflecting both inflammatory and adaptive responses. The application of a semi-quantitative scoring system enabled objective comparison between groups and is consistent with internationally accepted approaches to toxicological histopathological evaluation [60].

## 4. Materials and Methods

Extraction of CO_2_ under subcritical conditions.

The subcritical CO_2_ extraction of biologically active compounds from the rhizomes of sweet flag (*Acorus calamus* L.) and the flowers of marigold (*Calendula officinalis* L.) was carried out using a fluid extraction system under conditions ensuring that carbon dioxide remained in a liquid (subcritical) state. The extraction process was performed at a pressure of 6.0 MPa and a temperature of 22 °C, which allowed the preservation of thermolabile and volatile constituents typical of essential oil-bearing medicinal plants.

The plant material was pre-dried to an air-dry state and milled to a particle size of 1–3 mm. Extraction was conducted in a static-dynamic mode: the static phase lasted 15–30 min to ensure sufficient saturation of the plant matrix with CO_2_, followed by a dynamic extraction phase lasting 240 min.

The CO_2_ flow rate was maintained at 1–3 mL/min (or an equivalent value depending on the specifications of the equipment used). To ensure process reproducibility, identical extraction parameters were applied to both plant materials.

The obtained CO_2_ extracts were collected in light-protected containers and stored at +4 °C until further use in chemical (GC-MS) and pharmacological investigations.

GC-MS analysis conditions: sample volume 0.5 µL, injection temperature 280 °C, no split. Separation was performed using a 30 m DB-5MS capillary chromatographic column with an internal diameter of 0.25 mm and a film thickness of 0.25 µm at a constant carrier gas (helium) flow rate of 1 mL/min. The chromatographic temperature was programmed from 40 °C (hold 0 min) at a heating rate of 5 °C/min to 200 °C (hold 0 min), then at a heating rate of 10 °C/min to 280 °C (hold 1 min). Analysis time: 41 min. Detection was performed in SCAN mode (*m*/*z* 34–850). Agilent MSD ChemStation software (version 1701EA) was used to control the gas chromatography system and to record and process the results and data obtained (Agilent Technologies, Santa Clara, CA, USA).

Data processing included the determination of retention times and peak areas, as well as the processing of spectral information obtained using the mass spectrometry detector. The Wiley 7th edition and NIST’02 libraries were used to interpret the mass spectra obtained (the total number of spectra in the libraries exceeds 550,000).

The experimental studies were conducted at the animal facility of the B.A. Atchabarov Research Institute. Non-clinical trials were carried out in accordance with generally accepted methodological guidelines for experimental pharmacology, including R.U. Khabriev’s Guide to the Experimental (Preclinical) Study of New Pharmacological Substances (2005), as well as the methodological guidelines by K.A. Abdullin, K.D. Rakhimov, and Z.K. Kulzhanov on the evaluation of the antitussive activity of pharmacological substances (1997).

The following parameters were assessed during the study: acute toxicity, chronic toxicity, local irritant effect, allergenic properties, and the antitussive activity of the CO_2_ extracts under investigation, as well as of the newly developed tablet provisionally named ‘Exkair’.

### 4.1. Preparation of Plant CO_2_ Extract

*Calendula officinalis* L. was collected in August 2023 in the Sarkand District of the Zhetysu Region. The area is located in the foothills of the Dzungarian Alatau, at the peasant farm “Dary Prirody” (“Gifts of Nature”). *Acorus calamus* L. was collected in September 2023 in the Almaty Region, in the vicinity of Taldykorgan.

**Figure 5:**
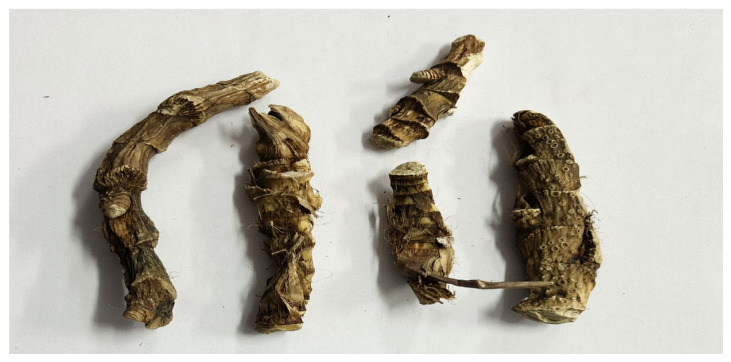
Rhizomes and roots of *Acorus calamus* L.

**Figure 6:**
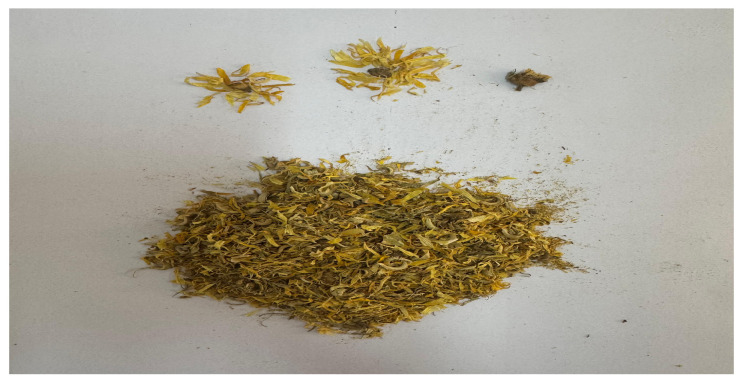
*Calendula officinalis* L. flowers.

Ground plant material from sweet flag and marigold was extracted using carbon dioxide extractors. This study investigated a concentrated CO_2_ extract of the rhizomes of sweet flag (*Acorus calamus* L., Figure 5) and flowers (*Calendula officinalis* L., Figure 6) obtained by low-temperature subcritical extraction with liquefied carbon dioxide (pressure 70 atm, temperature up to 30.5 °C) from air-dried raw materials.

Extraction conditions: UUPE extractor 5 I, particle size distribution 0.16–0.20 mm, bulk density 320 g/dm^3^, extraction duration 240 min, CO_2_ extract yield relative to raw material 4.0–4.2%, water consumption during extraction 10 L (circulation system). The rhizomes of marsh calamus are dense, so up to 5 kg of raw material was loaded into the unit. Based on the mass, 0.2 kg of CO_2_ extract was obtained.

To assess safety in experimental animals, the CO_2_ extract was dissolved in purified water to the required dose concentration and administered orally to mice at doses of 300, 500, 900, and 2500 mg/kg body weight. The resulting aqueous solutions of the CO_2_ extracts were administered orally using a specialised gastric tube.

### 4.2. Experimental Animals and Ethical Approval

The studies were conducted on laboratory animals, including white inbred mice weighing 18–25 g, rabbits, and guinea pigs. The animals were housed under standard vivarium conditions with controlled temperature, a natural light cycle, and free access to food and water. They were obtained from the Atchabarov Institute at the S.D. Asfendiyarov Kazakh National Medical University, Kazakhstan.

The animals were maintained in an animal facility with controlled environmental conditions at a temperature of 25 ± 1 °C and relative humidity of 60%, with a 12 h light-dark cycle, and had unlimited access to standard feed and drinking water. All animals were acclimated for one week prior to the experiment.

The study protocol was reviewed and approved by the Local Ethics Committee of the S.D. Asfendiyarov Kazakh National Medical University. S.D. Asfendiyarov Kazakh National Medical University Local Ethical Commission (LEC) Meeting No.8 (144) EXCERPT FROM THE MINUTES Meeting date: 3 November 2023.

### 4.3. Acute Toxicity Study

Acute toxicity was determined in white mice weighing 18–25 g. The aqueous solutions of CO_2_ extracts under investigation were administered orally on an empty stomach using a special probe at doses of 300, 500, 900 and 2500 mg/kg. Each dose was administered to mice in two series, each consisting of 10 groups; each test group comprised 6 mice.

The total number of experimental animals was 60. Every two hours following administration of the CO_2_ extracts (until the end of the working day), the test mice were observed for the presence or absence of symptoms of poisoning. The next observation was carried out on the 14th day. During the experiment, the following parameters were assessed: respiratory rate and depth, drowsiness, agility, coordination of movements, changes in the colour of the skin on the ears and tail, water and food intake, changes in body weight, frequency of urination, volume and consistency of faeces, and reaction to sound and light stimuli.

During the experiments, an assessment was carried out of the acute toxicity of CO_2_ extracts from the rhizomes of *Acorus calamus* L. and the flowers of *Calendula officinalis* L. The CO_2_ extracts under investigation were administered orally to laboratory animals at doses of 300, 500, 900 and 2500 mg/kg body weight.

### 4.4. Chronic Toxicity Study

The chronic toxicity of CO_2_ extracts of *Acorus calamus* rhizomes and *Calendula officinalis* flowers was determined by their oral administration over a period of 30 days. The study was conducted on white mice with a body weight of 18–25 g. Aqueous solutions of the test pharmacological substances were administered orally as suspensions via a special probe once daily for 30 days. The test animals were divided into two series, each comprising 5 groups. Each group consisted of 6 white mice.

Each series of animals included one control group and four experimental groups. In the first series, the control group of animals received purified water; the remaining four groups were administered oral suspensions of aqueous CO_2_ extracts of *Acorus calamus* rhizomes at doses of 300, 500, 900, and 2500 mg/kg, respectively. In the second series, the control group of animals received purified water; the remaining four groups were administered an aqueous solution of CO_2_ extract of *Calendula officinalis* L. flowers orally at doses of 300, 500, 900, and 2500 mg/kg, respectively.

The assessment of the chronic toxicity of CO_2_ extracts lasted 4 weeks, during which the test animals were observed every 2 h and at the end of the working day. During the observation, the following were assessed: respiratory rate and depth, drowsiness, speed and coordination of movements, changes in the colour of the skin on the ears and tail, water and food intake, changes in body weight, frequency of urination, volume and consistency of faeces, and reactions to auditory and visual stimuli. The body weight of the animals was measured once a week.

### 4.5. Assessment of Allergenic Properties

The allergenic properties of the test preparations were evaluated using standard pharmacological tests designed to detect hypersensitivity to the administered substances. Skin reactions were assessed, including the presence of oedema, hyperaemia, and other signs of an allergic response.

Assessment of allergenic potential is an important stage in the non-clinical testing of medicinal products. During the experiment, the development of possible hypersensitivity reactions following administration of the test preparations was monitored.

For the study of allergic reactions to carbon dioxide CO_2_ extracts of marsh calamus and calendula, application and conjunctival methods were used. For the application test, guinea pigs weighing 300–400 g were divided into 2 series of 10 groups each, and 0.5 g of 300 mg, 500 mg, 900 mg, and 2500 mg CO_2_ extracts of marsh calamus and medicinal calendula were applied five times over two weeks.

An assessment scale was used to compare skin reactions. Assessment scale:

0—no reaction;

1—white-red erythema;

2—light red erythema;

3—red erythema;

4—erythema and skin inflammation.

During the study of allergic reactions, fatty solutions of the CO_2_ extracts were used. *Acorus calamus* and *Calendula officinalis* CO_2_ extracts at 300, 500, 900, and 2500 mg, with 2 drops of 0.0002, 0.0003, 0.0006, and 0.001 mg administered into the eyes of 8 experimental rabbits. The rabbits’ eyes were then visually examined after 15 min, 24 h, and 48 h. Vegetable fats were administered to the animals.

### 4.6. Assessment of Local Irritant Effects

Subcutaneous and subconjunctival methods were used to investigate the irritant effects of carbon dioxide CO_2_ extracts of calamus rhizomes and calendula flowers.

For the subcutaneous study, experiments were conducted on 30 guinea pigs, divided into 5 groups of 6 guinea pigs each. The body weight of each guinea pig was 270–350 g. During the experiment, purified water was used for the animals in group 1, whilst a carbon dioxide CO_2_ extract of calamus rhizomes was administered to the animals in groups 2, 3, 4, and 5. For the animals in group 1 of series 2, purified water was used; for groups 2, 3, 4, and 5, a CO_2_ extract of *Calendula officinalis* flowers was administered. During the study of irritant effects, therapeutic concentrations of 0.3%, 0.5% and 0.9%, and 0.25% solutions, which were administered subcutaneously into the back of the guinea pig at a dose of 0.5 mL. No irritant effects were observed. The skin condition was satisfactory. The animals were kept under observation for 3 days.

Using the subconjunctival method, 0.3%, 0.5%, 0.9%, and 0.25% ointment solutions of marsh calamus and calendula CO_2_ extracts were instilled, two drops at a time using a tube, into the right eye of eight rabbits. Purified water was instilled into the left eye. The rabbits’ eyes were then visually examined after 30 min, 4 h, and 24 h.

The local irritant effects of the preparations were assessed following topical application to the skin and mucous membranes of laboratory animals. During observation, the development of inflammatory reactions, the degree of hyperaemia, and the severity of tissue irritation were recorded.

### 4.7. Evaluation of Antitussive Activity

The antitussive activity of the CO_2_ extracts and the tablet form, provisionally named ‘Exkair’, was studied in experimental models of inflammation. The efficacy of the preparations was assessed based on the degree of reduction in the severity of the inflammatory process and the dynamics of regression of inflammatory changes.

Components per one tablet (0.6 g):

CO_2_ extract of *Acorus calamus* (calamus rhizome)—0.009 g.

CO_2_ extract of *Calendula officinalis* flowers—0.003 g.

Ascorbic acid—0.005 g.

Calcium stearate—0.006 g.

Aerosil (colloidal silicon dioxide)—0.006 g.

Sugar—0.571 g.

Method of tablet preparation by compression using wet granulation.

Composition per granule:

Thick CO_2_ extract of Acorus calamus rhizomes—0.025 g.

Thick CO_2_ extract of Calendula officinalis flowers—0.015 g.

Ascorbic acid—0.003 g.

Excipients:

Icing sugar—3.000 g.

Citric acid—0.003 g.

Total mass: 3.046 g.

The granules were prepared using the wet granulation method, which is suitable for ingredients that are stable to moisture and subsequent drying. The granulation mass was mixed for 10 min. until a uniform moistened mass was obtained. The resulting wet mass was granulated using an “Fl-600” granulator with the addition of citric acid, after which it was dried in a drying oven at 50 °C for 1.5–2 h. The obtained granules were sieved through a screen with a 3 mm aperture diameter. Ascorbic acid was added to the dried granules and thoroughly mixed.

Prior to the start of the experiment, the laboratory animals were subjected to food deprivation for 14–16 h. The study involved 12 guinea pigs of both sexes (6 males and 6 females) weighing 300–400 g. Each animal was placed individually in plexiglass chambers measuring 20 × 14 × 12 cm.

Citric acid aerosol was used to induce the cough reflex. Using a pneumatic compressor, the animals were administered a 10–17% citric acid aerosol for 5 min. The experiment consisted of two consecutive stages.

In the first stage, conducted 24 h prior to the administration of the test substances, the individual sensitivity of the animals to citric acid was assessed. Animals exhibiting a pronounced coughing response were selected for further study. The experiment included guinea pigs in which an average of 20 to 30 coughing fits were recorded during a 30 min observation period.

In the second stage, conducted the following day, the pharmacological activity of the test preparations with expectorant action (tablet and granule dosage forms) was assessed. The preparations were administered to the animals enterally into the stomach using a special probe 30–60 min prior to the induction of the cough reflex.

Following administration of the test substances, the animals were repeatedly exposed to a 10–17% citric acid aerosol for 5 min using a pneumatic compressor. Thereafter, the number of coughing fits was recorded every 15–20 min.

A persistent and chronic cough is the main clinical manifestation of inflammatory processes in the respiratory tract. In clinical practice, the combined use of antitussive and anti-inflammatory agents is considered an effective approach. However, the prolonged use of antitussive agents alone often fails to provide sufficient therapeutic efficacy, necessitating the search for additional pharmacological agents to enhance the therapeutic outcome.

A key objective of contemporary pharmacological research is the development and introduction of new medicinal substances capable of effectively suppressing coughs arising from various respiratory tract pathologies. To address this challenge, an experimental model of airway inflammation was established in laboratory animals to evaluate the efficacy of standard and proprietary antitussive preparations, including ‘ZERP-AK BRONCHO’ granules and ‘Exkair’ tablets.

The study was conducted over a period of 3–4 months on male guinea pigs weighing 270–320 g. The animals were housed under standard vivarium conditions and received their usual diet. To prevent the gag reflex and the risk of aspiration, feeding was suspended 14–16 h prior to the experiment.

Each animal was placed in an individual plexiglass chamber measuring 20 × 14 × 126 cm. To simulate the cough reflex, an aerosol of a 10–17% aqueous citric acid solution was delivered into the chamber for 5 min using an ultrasonic pneumatic compressor. The day before the experiment, each animal was tested for sensitivity to citric acid to rule out possible allergic reactions.

The experimentally induced cough was repeated in the animals on average 20–30 times over a 30 min period. In the subsequent stage of the study, treatment was administered: the animals were given the standard antitussive drug Libexin and the author’s proprietary preparations under investigation. The preparations were administered in various dosages 5–10 min prior to cough stimulation. The control group of animals was kept under standard conditions without exposure to any medicinal products (see Table 8, Table 9 and Table 10).

All animals were divided into eight experimental groups, of which groups I and II served as controls.

### 4.8. Histopathological Scoring System

Histopathological changes were evaluated using a semi-quantitative scoring system:

0—no changes;

1—mild changes;

2—moderate changes;

3—severe changes.

The following parameters were assessed: epithelial oedema, epithelial hyperplasia, inflammatory cell infiltration, peribronchial inflammation, cartilage damage, and vascular congestion.

### 4.9. Statistical Data Analysis

Statistical analyses were performed using one-way analysis of variance (ANOVA) followed by Tukey’s multiple comparison test. The normality of data distribution was assessed using the Shapiro–Wilk test.

## 5. Statistical Analysis

All data are expressed as mean ± standard error of the mean (SEM).

A value of *p* < 0.05 was considered statistically significant.

No statistically significant differences in body weight were observed between the control and treated groups (300, 500, 900, and 2500 mg/kg) at any time point (Day 0, Day 7, and Day 14) (*p* > 0.05). Body weight increased progressively in all groups throughout the experimental period.

These findings indicate that administration of the test substance at the studied doses did not significantly affect body weight gain, suggesting the absence of systemic toxicity affecting general metabolic status.

All data are presented as mean ± standard error of the mean (SEM). Statistical analysis was performed using one-way analysis of variance (ANOVA) followed by Tukey’s multiple comparison test. The normality of data distribution was assessed using the Shapiro–Wilk test.

A value of *p* < 0.05 was considered statistically significant.

The analysis revealed a statistically significant decrease in the volume of inflammatory oedema in all treated groups compared to the control group (*p* < 0.05). The most pronounced anti-inflammatory effect was observed in the group receiving tablets under the provisional name “Exkair”, followed by calendula and calamus CO_2_ extracts.

The degree of inflammation suppression was 32.7% for calamus extract, 37.9% for calendula extract, and 46.5% for “Exkair” tablets, confirming their pharmacological activity. These results indicate that all tested samples exhibit significant anti-inflammatory effects, with the combined formulation demonstrating the highest efficacy.

## 6. Conclusions

A comprehensive evaluation of the experimental data indicates that the CO_2_ extracts of *Acorus calamus* rhizomes and *Calendula officinalis* flowers exhibit a favourable safety profile along with pronounced biological activity.

Non-clinical studies demonstrated the absence of signs of acute toxicity following administration of high doses of the test samples, classifying them as substances with a low level of toxicity according to generally accepted classifications. The results indicate good tolerability of the preparations and the absence of clinically significant changes in behavioural, physiological, or morphological parameters in experimental animals. These findings are of fundamental importance for the development of new herbal medicines, where a favourable safety profile is a key criterion during pharmaceutical development and non-clinical evaluation.

Pharmacological studies further demonstrated pronounced antitussive activity in the developed tablet form, provisionally named ‘Exkair’, and the granule form, provisionally named ‘ZERP-AK-BRONCHO’. A statistically significant reduction in inflammatory response indicators (*p* < 0.05) was observed compared with the control group, confirming the pharmacological efficacy of the formulations. This effect is likely attributable to the presence of a complex of biologically active compounds, including terpenoids, flavonoids, and phenolic compounds, which possess established anti-inflammatory properties.

Particular attention should be given to the use of a combined approach based on a mixture of CO_2_ extracts from various medicinal plants. The observed pharmacological effect is likely attributable to the synergism of biologically active substances, realised through a multifactorial impact on key components of the inflammatory process, including inhibition of inflammatory mediators, antioxidant activity, and stabilisation of cell membranes. These mechanisms align with current understanding of multi-component phytotherapy and are supported by the literature.

The results obtained provide a scientific rationale for further in-depth research, including extended non-clinical trials, standardisation of composition, investigation of mechanisms of action, and development of rational dosage forms. The combination of the identified pharmacological and toxicological properties supports the continued pharmaceutical development of medicinal products based on CO_2_ extracts of *Acorus calamus* L. and *Calendula officinalis* L., with a view to their subsequent introduction into medical practice in the Republic of Kazakhstan.

## Figures and Tables

**Table 2:** Results of the acute toxicity study of carbon dioxide (CO_2_) plant extracts in white mice.

Substance Concentration (mg/kg)	Substance Name					
	Control Group		*Acorus calamus* Rhizome CO_2_ Extract		*Calendula officinalis* Flower CO_2_ Extract	
	l	d	l	d	l	d
300	6	0	6	0	6	0
500	6	0	6	0	6	0
900	6	0	6	0	6	0
2500	6	0	6	0	6	0

Note: l—live; d—dead animals.

**Table 3:** Results of the chronic toxicity study of carbon dioxide (CO_2_) plant extracts in white mice.

Substance Quantity (mg/kg)	Name of Substance					
	Control Group (Purified Water)		CO_2_ Extract of Calamus Rhizomes		CO_2_ Extract of Calendula Flower	
	l	d	l	d	l	d
300	6	0	6	0	6	0
500	6	0	6	0	6	0
900	6	0	6	0	6	0
2500	6	0	6	0	6	0

Note: l—live; d—dead animals.

**Table 4:** Changes in body weight in white mice after 4 weeks of administration of the test substance (expressed as percentage of initial body weight).

Animal Group	Duration of the Experiment (Weeks)				
	0	1	2	3	4
1—control (purified water)	21 ± 1.03	22 ± 1.31	22 ± 0.60	23 ± 0.86	20 ± 0.58
2—receiving calamus rhizome CO_2_ extract	22 ± 0.58 (*p* > 0.05)	22 ± 1.24 (*p* > 0.05)	22 ± 1.05 (*p* > 0.05)	18 ± 0.58 (*p* < 0.01)	19 ± 0.36 (*p* > 0.05)
3—containing calendula flower CO_2_ extract	22 ± 1.24 (*p* > 0.05)	22 ± 1.05 (*p* > 0.05)	20 ± 0.86 (*p* > 0.05)	18 ± 0.91 (*p* < 0.01)	19 ± 0.68 (*p* > 0.05)

**Table 5:** Haematological parameters of white mice in the chronic toxicity study.

Parameter Measured	Animal Groups				
	1	2	3	4	5
Week 1					
Red blood cells (×10^12^/L)	8.83 ± 0.02	8.9 ± 0.10	8.8 ± 0.30	8.8 ± 0.40	8.74 ± 0.01
Haemoglobin (g/L)	14.3 ±14.6	14.6 ± 30	15.0 ± 50	14.0 ± 40	13.8 ± 5.9
White blood cells (×10^9^/L)	6.55 ± 0.67	6.8 ± 0.60	6.4 ± 0.60	6.5 ± 0.90	6.14 ± 0.53
Week 4					
Red blood cells	8.80 ± 0.04	9.2 ± 0.20	8.0 ± 0.10	8.5 ± 0.20	8.76 ± 0.04
Haemoglobin	14.2 ± 5.80	14.9 ± 20	15.7 ± 30	14.9 ± 30	14.2 ± 7.30
White blood cells	6.97 ± 0.72	7.7 ± 0.40	6.6 ± 0.80	6.9 ± 0.40	6.31 ± 0.55

**Table 6:** Internal organ weights in white mice after CO_2_ extract administration for chronic toxicity assessment.

Organs Studied	Animal Groups				
	1	2	3	4	5
Heart	1.12 ± 0.63	1.02 ± 0.23	1.14 ± 0.21	1.10 ± 0.42	1.97 ± 0.72
Liver	1.32 ± 1.18	1.52 ± 0.18	1.42 ± 0.28	1.38 ± 0.23	1.60 ± 1.07
Kidneys	0.72 ± 0.48	0.63 ± 0.38	0.70 ± 0.33	0.67 ± 0.34	0.63 ± 0.42
	0.74 ± 0.42	0.64 ± 0.34	0.72 ± 0.22	0.70 ± 0.14	0.65 ± 0.39
Lungs	0.67 ± 0.10	0.62 ± 0.12	0.66 ± 0.20	0.63 ± 0.20	0.64 ± 0.09

**Table 7:** Antitussive activity of the investigated drugs expressed as a percentage.

Drugs	Doses			
	3 mg/kg	5 mg/kg	9 mg/kg	25 mg/kg
Exkair	1.22%	14.16%	31.42%	57.27%

**Table 8:** Correlation coefficients of antitussive activity for Exkair, Libexin, and Mucaltin.

Preparations	M ± m			
	Doses			
	3 mg/kg	5 mg/kg	9 mg/kg	25 mg/kg
Exkair	2.73 ± 0.32 *p* < 0.05	3.15 ± 0.33 *p* < 0.001	2.15 ± 0.2 *p* < 0.001	8.56 ± 0.16 *p* < 0.05
Libexin	3.84 ± 0.26	8.0 ± 0.17	8.73 ± 0.007	6.96 ± 0.16
Mucaltin	2.5 ± 0.30 *p* < 0.05	3.01 ± 0.30 *p* < 0.001	2.02 ± 0.28 *p* < 0.001	7.45 ± 0.12 *p* < 0.05
	*p*_1_ < 0.05 *p*_2_ < 0.05	*p*_1_ < 0.001 *p*_2_ < 0.001	*p*_1_ < 0.001 *p*_2_ < 0.001	*p*_1_ < 0.05 *p*_2_ < 0.05

Note: *p*_1_—with Libexin; *p*_2_—correlation coefficient compared with Mucaltin.

**Table 9:** Comparative evidence of the antitussive efficacy of ZERP-AK BRONCHO granules and the reference drug.

Preparations	M ± m			
	Doses			
	3 mg/kg	5 mg/kg	9 mg/kg	25 mg/kg
‘ZERP-AK BRONCHO’	1.96 ± 0.1	15.17 ± 0.33	32.92 ± 0.2	58.37 ± 0.16
Libexin	41.85 ± 0.26	35.83 ± 0.17	30.55 ± 0.007	24.19 ± 0.16
Mucaltin	1.09 ± 0.30	13.96 ± 0.30	30.01 ± 0.28	36.88 ± 0.12
	*p*_1_ < 0.001 *p*_2_ < 0.05	*p*_1_ < 0.05 *p*_2_ > 0.05	*p*_1_ > 0.05 *p*_2_ < 0.05	*p*_1_ < 0.01 *p*_2_ < 0.05

Note: *p*_1_—with Libexin; *p*_2_—correlation coefficient compared with Mucaltin.

**Table 10:** Histopathological semi-quantitative assessment.

Figures	Oedema	Infiltration	Hyperplasia	Structural Damage
Figure S6	2	1	0	2
Figure S7	2	2	1	2
Figure S8	2	2	1	2
Figure S9	2	2	0	1
Figure S12	2	2	1	1
Figure S14	1	2	2	1
Figure S15	1	2	1	1
Figure S16	1	1	0	0
Figure S17	3	2	0	2
Figure S18	1	1	0	1
Figure S19	3	0	0	3
Figure S20	2	2	0	1
Figure S21	3	2	0	3

**Table 11:** Comparative antitussive efficacy of Exkair and standard agents.

Drug	3 mg/kg	5 mg/kg	9 mg/kg	25 mg/kg
Exkair	1.22%	14.16%	31.42%	57.27%
Libexin	41.85%	35.83%	30.55%	24.19%
Mucaltin	1.09%	13.96%	30.01%	36.88%

## Data Availability

The original contributions presented in this study are included in the article/Supplementary Materials. Further inquiries can be directed to the corresponding author.

## Supplementary Materials

The following supporting information can be downloaded at: https://www.mdpi.com/article/10.3390/ph19050789/s1. Table S1. Results of GC-MS analysis of the CO_2_ extract of *Acorus calamus* L. Figure S1. Chromatogram of the CO_2_ extract of *Acorus calamus* L. Figure S2. Histological section of lung tissue from a white mouse treated with a carbon dioxide extract of *Acorus calamus* rhizomes. Figure S3. Histological section of kidney tissue from a white mouse treated with a carbon dioxide extract of *Acorus calamus* rhizomes (H&E staining). Figure S4. Histological section of liver tissue from a white mouse treated with a carbon dioxide extract of *Acorus calamus* rhizomes (haematoxylin and eosin staining). Figure S5. Histopathological structure of the bronchial walls in animals from the observation group: (A) large bronchi; (B) medium bronchi, ×100, haematoxylin and eosin staining. Figure S6. Histopathological changes in the large bronchus, including epithelial oedema, intercartilaginous oedema, and narrowing of the bronchial lumen, ×100, haematoxylin and eosin staining. Figure S7. Histopathological changes in the main bronchus of animals from the observation group, including focal epithelial desquamation, inflammatory cell infiltration, and deformation of the bronchial lumen (hourglass-shaped), ×100, haematoxylin and eosin staining. Figure S8. Histopathological changes in the bronchial wall, including epithelial infiltration, inflammatory involvement of the cartilage, ×100, haematoxylin and eosin staining. Figure S9. Histopathological changes in lung tissue characterised by interstitial pneumonitis and peribronchial inflammatory cell infiltration, ×100, haematoxylin and eosin staining. Figure S10. Histopathological changes in the large bronchi, including focal epithelial infiltration, peribronchial inflammatory cell infiltration with oedema, and chondrocyte dystrophy, ×100, haematoxylin and eosin staining. Figure S11. Histopathological changes in the middle bronchus of animals in Group 5 (Libexin-treated group, 9 mg), showing focal epithelial hyperplasia, ×100, haematoxylin and eosin staining. Figure S12. Histopathological changes characterised by focal interstitial pneumonitis and epithelial alterations in the bronchial wall, ×100, haematoxylin and eosin staining. Figure S13. Histopathological changes in large bronchi, including pronounced peribronchial inflammatory cell infiltration and deformation of the bronchial lumen, ×100, haematoxylin and eosin staining. Animals of Group 6 were treated with the reference drug Libexin (20 mg). Figure S14. Histopathological changes in the bronchial wall, including epithelial hyperplasia and focal peribronchial inflammatory cell infiltration, ×100, haematoxylin and eosin staining. Figure S15. Histopathological changes in the large bronchus, including focal epithelial hyperplasia and peribronchial inflammatory cell infiltration, ×100, haematoxylin and eosin staining. Figure S16. Histopathological changes in the large bronchus, characterised by peribronchial inflammatory cell infiltration with preservation of the epithelial layer, ×100, haematoxylin and eosin staining. Figure S17. Histopathological changes in lung tissue and small bronchi, including wall oedema and vascular congestion (blood-filled vessel), ×200, haematoxylin and eosin staining. Figure S18. Histopathological changes in the large bronchus, showing mild wall oedema and partially preserved inflammatory focus, ×100, haematoxylin and eosin staining. Animals in Group 7a were treated with 9 mg granules of the investigational compound. Figure S19. Histopathological changes in the large bronchus, showing tumour formation in the bronchial wall, ×100, haematoxylin and eosin staining. Figure S20. Histopathological changes in lung tissue characterised by interstitial pneumonitis, ×100, haematoxylin and eosin staining. Figure S21. Histopathological changes in the airways, including oedema of the large bronchial walls and peribronchial sclerosis in the medium bronchi, ×100, haematoxylin and eosin staining.
