# Supplementary material for: Assessing the Safety of Carbon Dioxide Extracts of Acorus calamus Rhizomes and Calendula officinalis Flowers and the Antitussive Activity of the Tablet Dosage Form ‘Exkair’ and Granules ‘Zerp-Ak-Broncho’ Developed on Their Basis
Source: Pharmaceuticals (Basel). 2026 May 18;19(5):789. doi: 10.3390/ph19050789 (PMC13209801; doi:10.3390/ph19050789)
Supplement: Supplementary file 1 [file pharmaceuticals-19-00789-s001.zip › File S2.pdf]

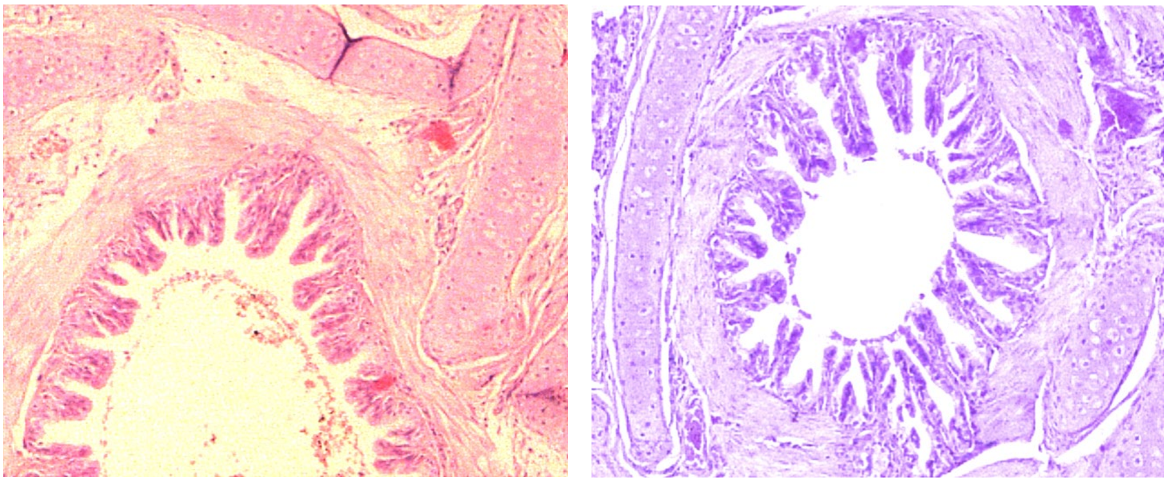

(A) (B)

Figure S5. Histopathological structure of the bronchial walls in animals from the observation group: (A) large bronchi; (B) medium bronchi, ×100, haematoxylin and eosin staining.

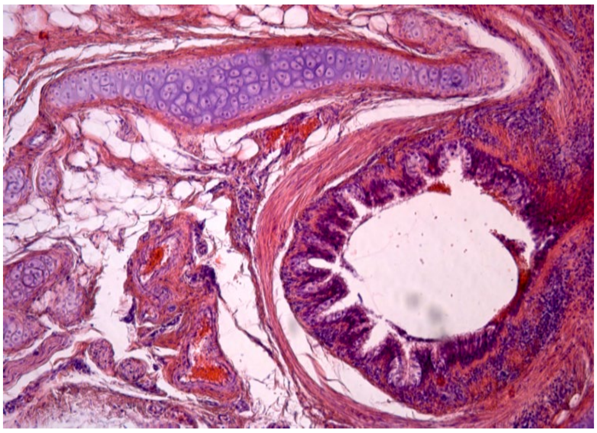

Figure S6. Histopathological changes in the large bronchus, including epithelial oedema, intercartilaginous oedema, and narrowing of the bronchial lumen, ×100, haematoxylin and eosin staining.

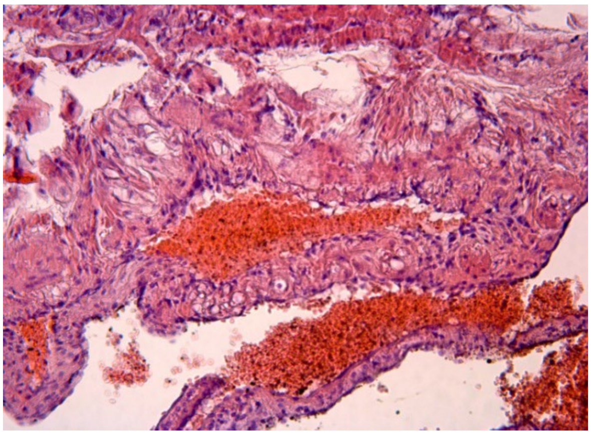

Figure S7. Histopathological changes in the main bronchus of animals from the observation group, including focal epithelial desquamation, inflammatory cell infiltration, and deformation of the bronchial lumen (hourglass-shaped), ×100, haematoxylin and eosin staining.

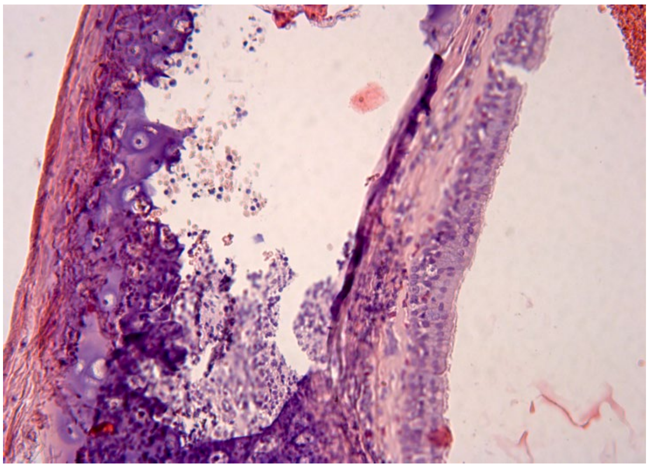

Figure S8. Histopathological changes in the bronchial wall, including epithelial infiltration, inflammatory involvement of the cartilage, ×100, haematoxylin and eosin staining.

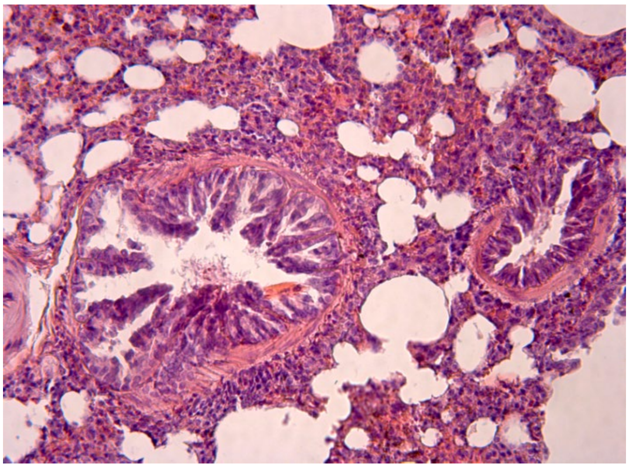

Figure S9. Histopathological changes in lung tissue characterised by interstitial pneumonitis and peribronchial inflammatory cell infiltration, ×100, haematoxylin and eosin staining.

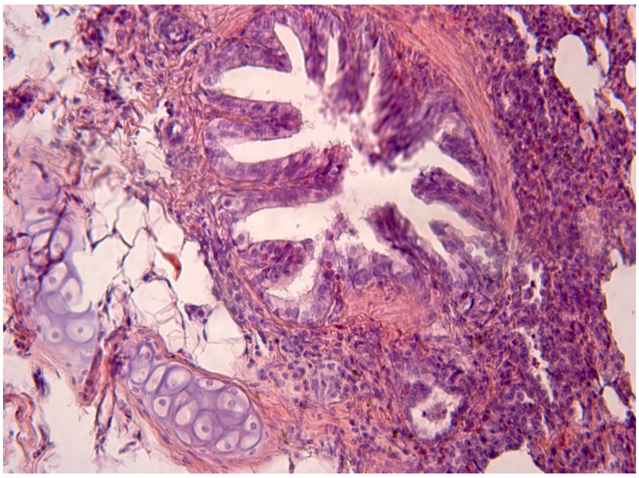

Figure S10. Histopathological changes in the large bronchi, including focal epithelial infiltration, peribronchial inflammatory cell infiltration with oedema, and chondrocyte dystrophy, ×100, haematoxylin and eosin staining.

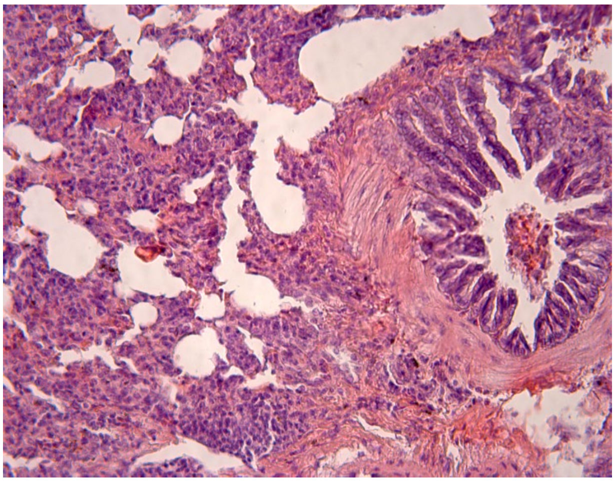

Figure S11. Histopathological changes in the middle bronchus of animals in Group 5 (Libexin-treated group, 9 mg), showing focal epithelial hyperplasia, ×100, haematoxylin and eosin staining.

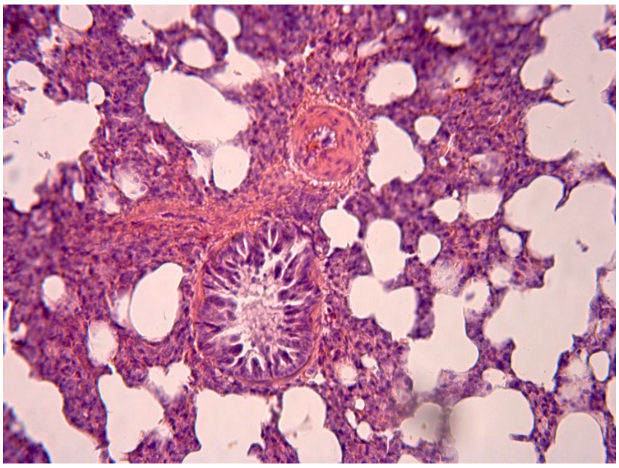

Figure S12. Histopathological changes characterised by focal interstitial pneumonitis and epithelial alterations in the bronchial wall, ×100, haematoxylin and eosin staining.

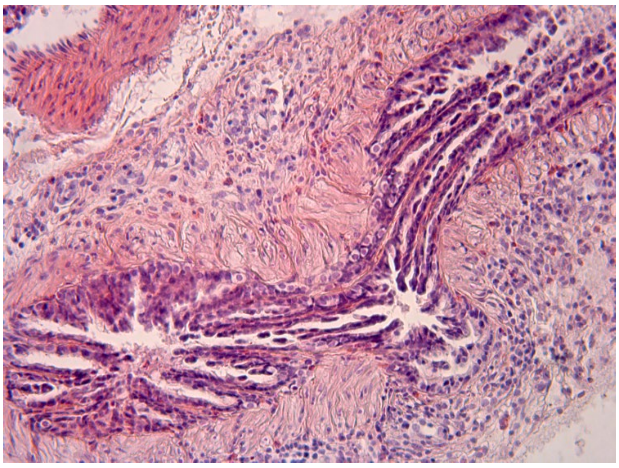

Figure S13. Histopathological changes in large bronchi, including pronounced peribronchial inflammatory cell infiltration and deformation of the bronchial lumen, ×100, haematoxylin and eosin staining. Animals of Group 6 were treated with the reference drug Libexin (20 mg).

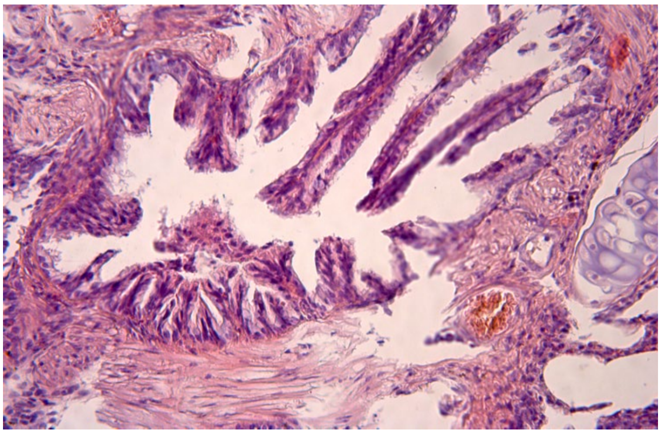

Figure S14. Histopathological changes in the bronchial wall, including epithelial hyperplasia and focal peribronchial inflammatory cell infiltration, ×100, haematoxylin and eosin staining.

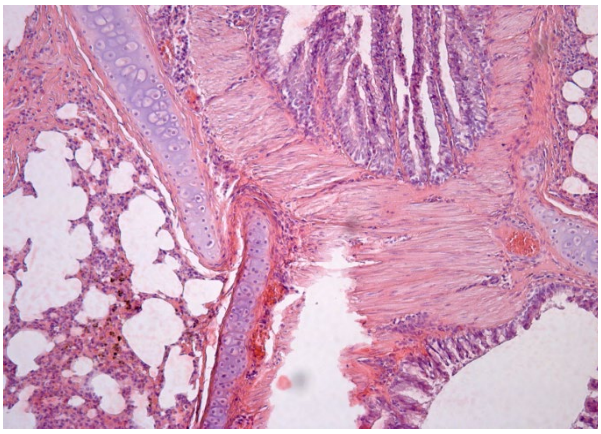

Figure S15. Histopathological changes in the large bronchus, including focal epithelial hyperplasia and peribronchial inflammatory cell infiltration, ×100, haematoxylin and eosin staining.

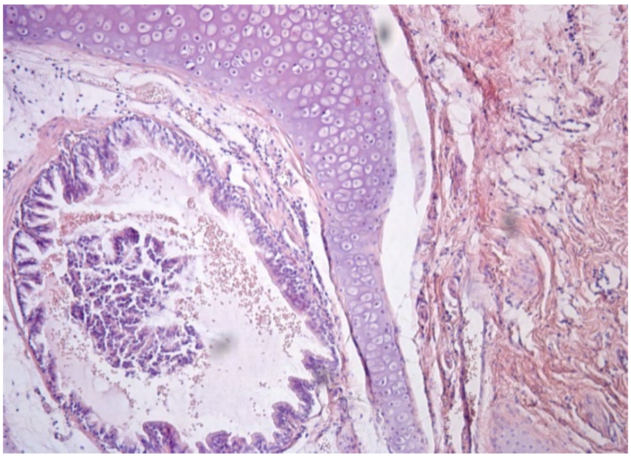

Figure S16. Histopathological changes in the large bronchus, characterised by peribronchial inflammatory cell infiltration with preservation of the epithelial layer, ×100, haematoxylin and eosin staining.

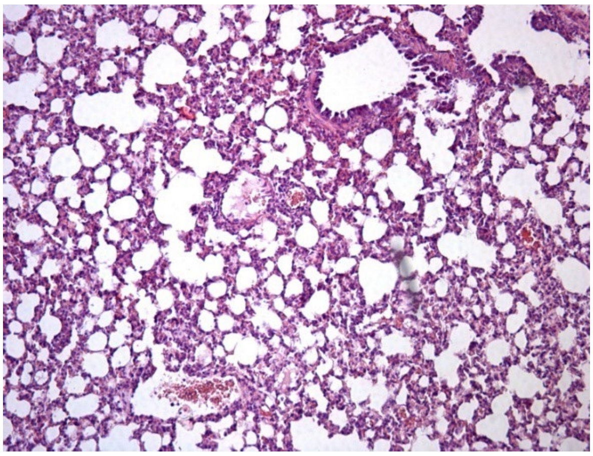

Figure S17. Histopathological changes in lung tissue and small bronchi, including wall oedema and vascular congestion (blood-filled vessel), ×200, haematoxylin and eosin staining.

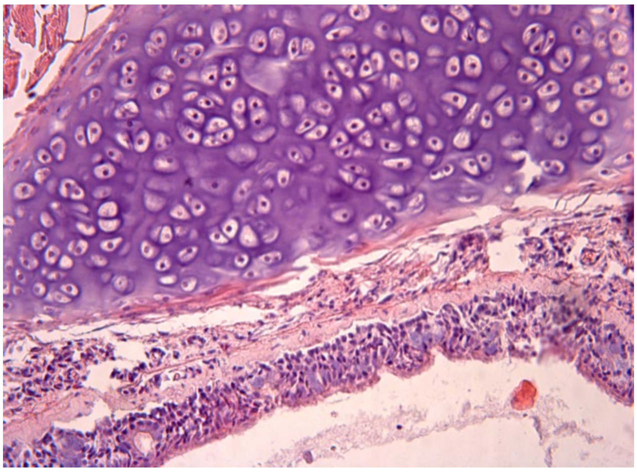

Figure S18. Histopathological changes in the large bronchus, showing mild wall oedema and partially preserved inflammatory focus, ×100, haematoxylin and eosin staining. Animals in Group 7a were treated with 9 mg granules of the investigational compound.

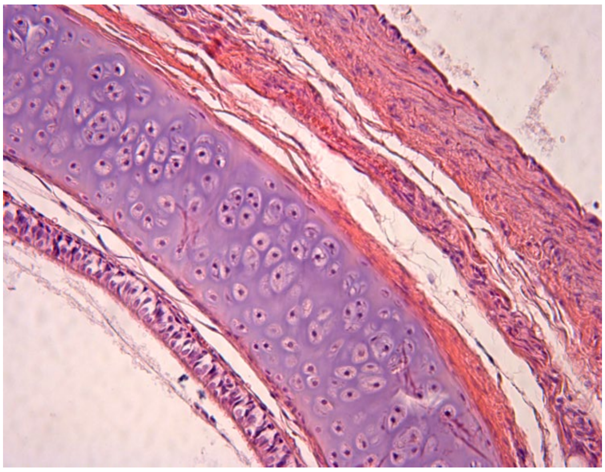

Figure S19. Histopathological changes in the large bronchus, showing tumour formation in the bronchial wall, ×100, haematoxylin and eosin staining.

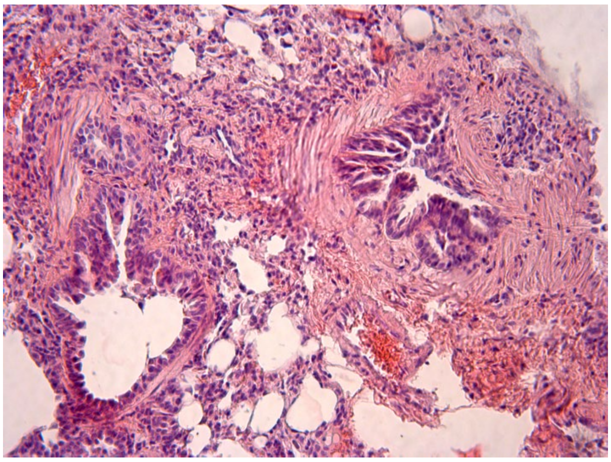

Figure S20. Histopathological changes in lung tissue characterised by interstitial pneumonitis, ×100, haematoxylin and eosin staining.

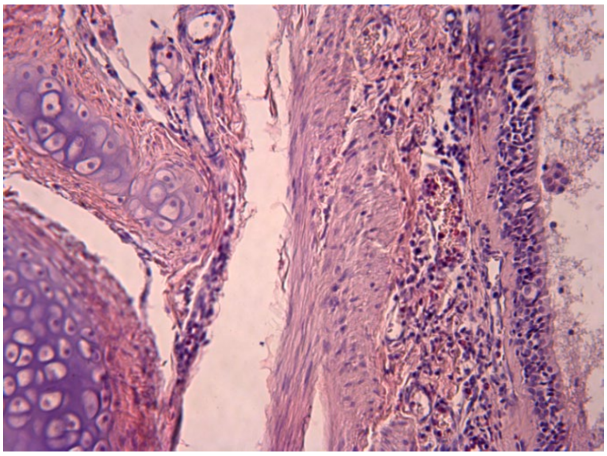

.

Figure S21. Histopathological changes in the airways, including oedema of the large bronchial walls and peribronchial sclerosis in the medium bronchi, ×100, haematoxylin and eosin staining.
