# Supplementary material for: Assessing the Safety of Carbon Dioxide Extracts of Acorus calamus Rhizomes and Calendula officinalis Flowers and the Antitussive Activity of the Tablet Dosage Form ‘Exkair’ and Granules ‘Zerp-Ak-Broncho’ Developed on Their Basis
Source: Pharmaceuticals (Basel). 2026 May 18;19(5):789. doi: 10.3390/ph19050789 (PMC13209801; doi:10.3390/ph19050789)
Supplement: Supplementary file 1 [file pharmaceuticals-19-00789-s001.zip › File S1.pdf]

Table S1. Results of GC-MS analysis of the CO_2 extract of *Acorus calamus* L.

№	Retention time, (min)	Compounds	Probability of identification, (%)	Percentage content, (%)
1	11.01	Diepoxybutane	79	0.24
2	12.21	Ethanoic acid	95	1.87
3	12.68	Methyl 2-oxopropanoate	88	1.96
4	14.14	Trimethylbicyclo[2.2.1]heptan-2-one	97	1.91
5	15.54	3,7-Dimethylocta-1,6-dien-3-ol	90	0.30
6	16.79	Cedr-8(15)-ene	83	0.49
7	17.40	Furan-2-ylmethanol	91	0.29
8	17.93	1,8a-Dimethyl-7-(prop-1-en-2-yl)octahydronaphthalene	84	0.68
9	18.93	Methyl 3,7-dimethylocta-2,6-dienoate	73	0.44
10	19.13	1,8-Dimethyl-4-(prop-1-en-2-yl)spiro[4.5]dec-7-ene	75	0.27
11	19.41	1-(1,5-Dimethylhex-4-en-1-yl)-4-methylbenzene	85	0.30
12	19.85	4a,8-Dimethyl-2-(prop-1-en-2-yl)octahydronaphthalene	88	2.01
13	20.70	1-(1,5-Dimethylhex-4-en-1-yl)-4-methylbenzene	87	0.15
14	21.57	1-(1,5-Dimethylhex-4-en-1-yl)-4-methylbenzene	86	0,35
15	21.91	3,7-Dimethylocta-2,6-dien-1-ol	89	0.36
16	22.80	Isoshyobunone	95	3.60
17	23.25	Shyobunone	92	4.70
18	23.39	α-Calacorene	84	0.67
19	24.28	9-Methoxycalamenene	78	0.43
20	24.68	4,11,11-Trimethyl-8-methylenebicyclo[7.2.0]undec-4-ene epoxide	85	0.51
21	24.99	4-Hydroxy-2,5-dimethylfuran-3(2H)-one	70	0.38
22	25.74	1,5,5,8-Tetramethyl-12-oxabicyclo[9.1.0]dodeca-3,7-diene	80	0.90

23	25.95	3,7,11-Trimethyldodeca-1,6,10-trien-3-ol	93	0.32
24	26.52	1,8-Dimethyl-4-(propan-2-yl)spiro[4.5]decan-7-one	86	1.54
25	26.69	Dehydroxy-isocalamendiol	89	1.39
26	27.20	Epicedrol	68	1.16
27	27.35	Spathulenol	87	2.45
28	27.42	2,3,4,4a,5,6,7,8-Octahydro-1,1,4a,7-tetramethyl-1H-benzocyclohepten-7-ol	67	0.52
29	27.91	2,4,6,7,8,8a-Hexahydro-3,8-dimethyl-4-(propan-2-ylidene)azulen-5-one	85	0.36
30	28.56	1,7-Dimethyl-4-(propan-2-yl)spiro[4.5]dec-6-en-8-one	82	12.78
31	28.77	3,5-Dihydroxy-2,3-dihydro-6-methyl-4H-pyran-4-one	88	1.40
32	29.17	Cadin-4-en-10-ol	89	0.36
33	29.48	Octahydro-2,2,4,7a-tetramethyl-1,3a-ethanoinden-4-ol	75	0.51
34	29.65	Propane-1,2,3-triol	67	1.01
35	30.71	4,8-Dimethyl-1-(propan-2-yl)spiro[4.5]dec-8-en-7-ol	88	1.32
36	30.95	1,2,4-Trimethoxy-5-(prop-1-en-1-yl)benzene	95	4.27
37	31.14	Murolan-3,9(11)-diene-10-peroxy	79	1.16
38	31.30	9-Methoxycalamenene	70	0.58
39	31.59	Widdrol hydroxyether	73	0.64
40	32.77	3,4,4a,5,6,8a-Hexahydro-2,5,5,8a-tetramethyl-2H-1-benzopyran	77	2.05
41	32.98	5-(Hydroxymethyl)furan-2-carbaldehyde	85	2.41
42	33.25	Isocalamendiol	81	11.15
43	33.47	1,1,5,5-Tetramethylhexahydro-2,4a-methanonaphthalen-7-one	79	0.58
44	33.95	2-o2-Methyl-2-(3-methyl-2-oxobutyl)cyclohexan-1-onexobutyl)	73	2.24
45	34.27	1-Heptanoyl-3-methylene-2-pentylcyclopropane	72	2.58
46	34.82	Perhydro-7,7,10a-trimethylbenzo[f]chromenetrimethyl	71	1.62

47	36.20	Isoaromadendrene epoxide	77	0.55
48	36.44	Diphenyl benzene-1,2-dicarboxylate	67	0.94
49	36.51	1-(3,4,5-Trimethoxyphenyl)propan-2-one	79	0.97
50	36.69	Isoaromadendrene epoxide	74	0.53
51	39.53	Hexadecanoic acid	91	3.78
52	40.26	2-Hydroxy-1,1,10-trimethyl-6,9-epidioxydecalin	73	1.09
53	43.27	Octadeca-9,12-dienoic acid	93	5.30
54	43.48	Glucopyranosyl-(1→2)-fructofuranoside	77	4.31
55	44.04	4-(2,6,6-Trimethylcyclohex-1-en-1-yl)butanoic acid	66	0.68
56	46.41	Ethyl glucopyranoside	84	2.49

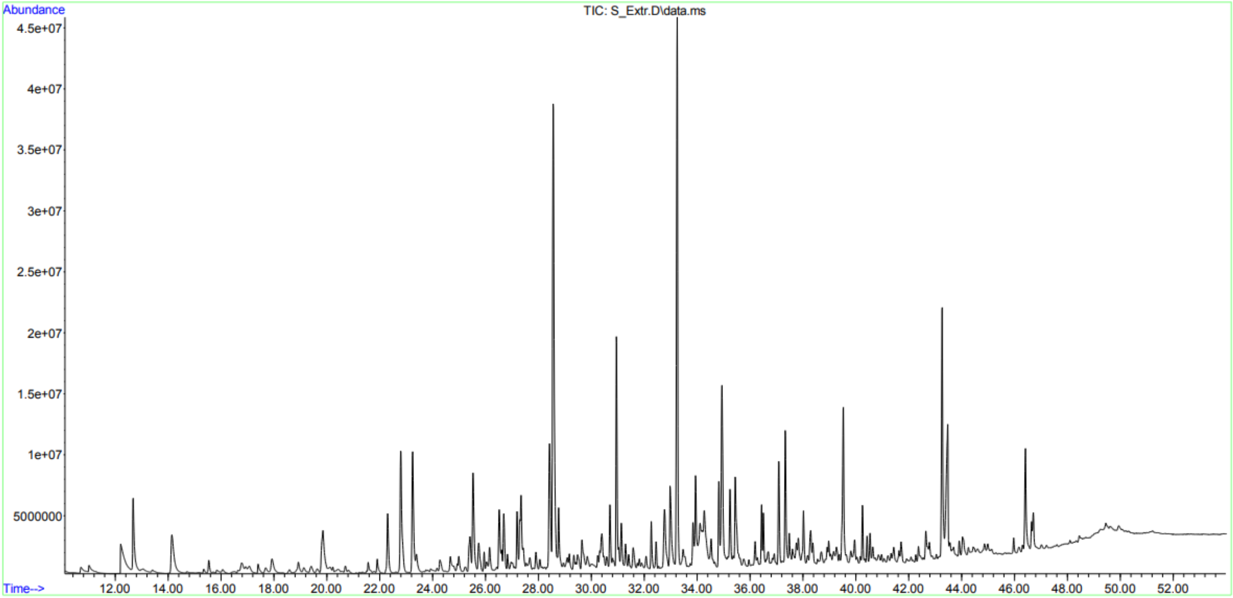

Figure S1. Chromatogram of the CO_2 extract of *Acorus calamus* L.

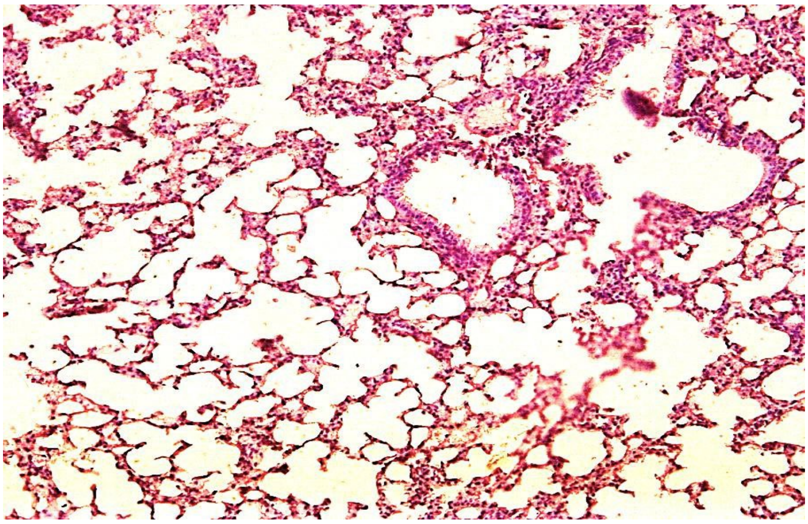

Figure S2. Histological section of lung tissue from a white mouse treated with a carbon dioxide extract of *Acorus calamus* rhizomes.

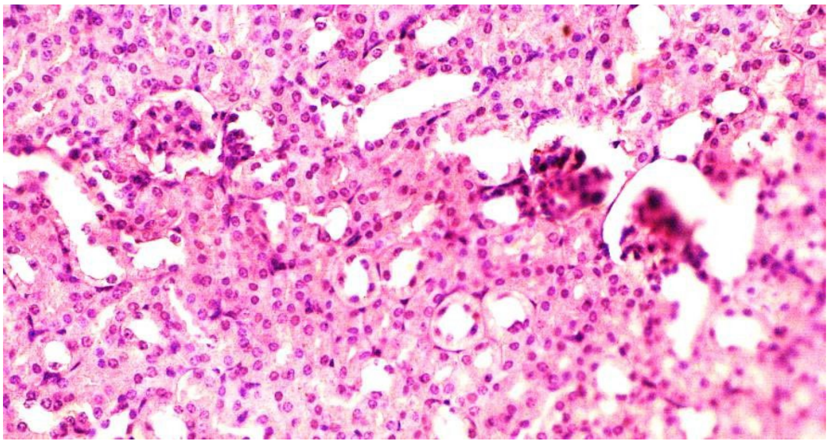

Figure S3. Histological section of kidney tissue from a white mouse treated with a carbon dioxide extract of *Acorus calamus* rhizomes (H&E staining).

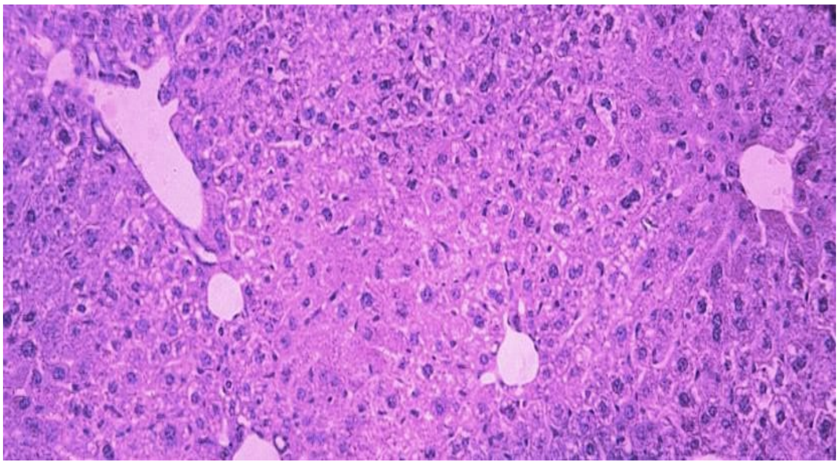

Figure S4. Histological section of liver tissue from a white mouse treated with a carbon dioxide extract of *Acorus calamus* rhizomes (haematoxylin and eosin staining).
